# Assessing atrophy measurement techniques in dementia: Results from the MIRIAD atrophy challenge

**DOI:** 10.1016/j.neuroimage.2015.07.087

**Published:** 2015-12

**Authors:** David M. Cash, Chris Frost, Leonardo O. Iheme, Devrim Ünay, Melek Kandemir, Jurgen Fripp, Olivier Salvado, Pierrick Bourgeat, Martin Reuter, Bruce Fischl, Marco Lorenzi, Giovanni B. Frisoni, Xavier Pennec, Ronald K. Pierson, Jeffrey L. Gunter, Matthew L. Senjem, Clifford R. Jack, Nicolas Guizard, Vladimir S. Fonov, D. Louis Collins, Marc Modat, M. Jorge Cardoso, Kelvin K. Leung, Hongzhi Wang, Sandhitsu R. Das, Paul A. Yushkevich, Ian B. Malone, Nick C. Fox, Jonathan M. Schott, Sebastien Ourselin

**Affiliations:** a Dementia Research Centre, UCL Institute of Neurology, London, UK; b Translational Imaging Group, Centre for Medical Image Computing, UCL, London, UK; c Department of Medical Statistics, London School of Hygiene and Tropical Medicine, London, UK; d Electrical & Electronics Engineering, Bahcesehir University, Istanbul, Turkey; e Biomedical Engineering, Bahcesehir University, Istanbul, Turkey; f Department of Neurology, Bayindir Hospital Icerenkoy, Istanbul, Turkey; g The Australian eHealth Research Centre, CSIRO Digital Productivity Flagship, Herston, QLD, Australia; h Martinos Center for Biomedical Imaging, Dept. of Radiology, Massachusetts General Hospital, Harvard Medical School, Boston, MA, USA; i Computer Science and Artificial Intelligence Lab, Massachusetts Institute of Technology, Cambridge, MA, USA; j Asclepios Research Project, INRIA Sophia Antipolis, Sophia Antipolis, France; k IRCCS San Giovanni di Dio Fatebenefratelli, Brescia, Italy; l Memory Clinic and Laboratoire de Neuroimagerie du Vieillissement (LANVIE), University Hospitals and University of Geneva, Geneva, Switzerland; m Brain Image Analysis, LLC, Coralville, IA, USA; n Department of Radiology, Mayo Clinic, Rochester, MN, USA; o McConnell Brain Imaging Centre, Montreal Neurological Institute, McGill University, Montreal, QC, Canada; p Almaden Research Center, IBM Research, Almaden, CA, USA; q Penn Image Computing and Science Laboratory, Department of Radiology, University of Pennsylvania, USA

## Abstract

Structural MRI is widely used for investigating brain atrophy in many neurodegenerative disorders, with several research groups developing and publishing techniques to provide quantitative assessments of this longitudinal change. Often techniques are compared through computation of required sample size estimates for future clinical trials. However interpretation of such comparisons is rendered complex because, despite using the same publicly available cohorts, the various techniques have been assessed with different data exclusions and different statistical analysis models. We created the MIRIAD atrophy challenge in order to test various capabilities of atrophy measurement techniques. The data consisted of 69 subjects (46 Alzheimer's disease, 23 control) who were scanned multiple (up to twelve) times at nine visits over a follow-up period of one to two years, resulting in 708 total image sets. Nine participating groups from 6 countries completed the challenge by providing volumetric measurements of key structures (whole brain, lateral ventricle, left and right hippocampi) for each dataset and atrophy measurements of these structures for each time point pair (both forward and backward) of a given subject. From these results, we formally compared techniques using exactly the same dataset. First, we assessed the repeatability of each technique using rates obtained from short intervals where no measurable atrophy is expected. For those measures that provided direct measures of atrophy between pairs of images, we also assessed symmetry and transitivity. Then, we performed a statistical analysis in a consistent manner using linear mixed effect models. The models, one for repeated measures of volume made at multiple time-points and a second for repeated “direct” measures of change in brain volume, appropriately allowed for the correlation between measures made on the same subject and were shown to fit the data well. From these models, we obtained estimates of the distribution of atrophy rates in the Alzheimer's disease (AD) and control groups and of required sample sizes to detect a 25% treatment effect, in relation to healthy ageing, with 95% significance and 80% power over follow-up periods of 6, 12, and 24 months. Uncertainty in these estimates, and head-to-head comparisons between techniques, were carried out using the bootstrap. The lateral ventricles provided the most stable measurements, followed by the brain. The hippocampi had much more variability across participants, likely because of differences in segmentation protocol and less distinct boundaries. Most methods showed no indication of bias based on the short-term interval results, and direct measures provided good consistency in terms of symmetry and transitivity. The resulting annualized rates of change derived from the model ranged from, for whole brain: − 1.4% to − 2.2% (AD) and − 0.35% to − 0.67% (control), for ventricles: 4.6% to 10.2% (AD) and 1.2% to 3.4% (control), and for hippocampi: − 1.5% to − 7.0% (AD) and − 0.4% to − 1.4% (control). There were large and statistically significant differences in the sample size requirements between many of the techniques. The lowest sample sizes for each of these structures, for a trial with a 12 month follow-up period, were 242 (95% CI: 154 to 422) for whole brain, 168 (95% CI: 112 to 282) for ventricles, 190 (95% CI: 146 to 268) for left hippocampi, and 158 (95% CI: 116 to 228) for right hippocampi. This analysis represents one of the most extensive statistical comparisons of a large number of different atrophy measurement techniques from around the globe. The challenge data will remain online and publicly available so that other groups can assess their methods.

## Highlights

• We compared numerous brain atrophy measurement techniques using multiple metrics.
• Each participant produced measures on the exact same dataset, blinded to disease.
• A central statistical analysis using linear mixed effect models was performed.
• Head to head comparisons for each region were performed using sample size estimates.
• Brain and ventricle measures were more consistent across groups than for hippocampi.

## Introduction

Alzheimer's disease (AD) presents significant challenges to health care systems throughout the world as the elderly population worldwide increases and no disease-modifying treatments are currently available. Accurate and robust measurements are needed to aid in diagnosis, to track disease progression, and to determine whether the underlying disease is being modified by a potential new therapy. Whilst the primary outcome measures for randomized controlled clinical trials of potential disease-modifying agents are likely to be cognitive outcomes, they often suffer from such issues as floor/ceiling effects, practice effects, and rater subjectivity that results in high variability (Black et al., 2009). It is also clear that there is a long (> 10 years) prodromal period of the disease, where cognitive deficits are minimal and subtle, but there are numerous changes that are observable through imaging techniques.

Rates of atrophy calculated from serial magnetic resonance imaging (MRI) are one of the most widely used imaging biomarkers in dementia and are increasingly considered as potential surrogates for disease activity, the treatment effect being the difference in the mean slopes between treated and placebo groups (Benzinger et al., 2013, Schott et al., 2010, Villemagne et al., 2013). These measurements are often more sensitive than cognitive measures, as illustrated by lower sample sizes of subjects per arm that would be required in order to be appropriately statistically powered (Jack et al., 2004). Compared to other imaging biomarkers e.g., positron emission tomography (PET) measures of amyloid deposition and hypometabolism, MRI based measures of atrophy are thought to provide better surrogates of disease progression, with changes beginning before, but very close to, clinical disease onset (Jack et al., 2013). Numerous techniques exist to compute atrophy from longitudinal MRI scans of the brain, including (Freeborough and Fox, 1997, Holland and Dale, 2011, Hua et al., 2013, Reuter et al., 2012). Many of these methods have been applied to the same set of large, publicly available cohorts, and in some cases, comparisons between different methods have been performed, primarily by comparing the effect sizes of each method through the estimation of sample sizes needed to statistically power a hypothetical clinical trial.

For the most part, direct comparisons between multiple techniques using exactly the same dataset have not yet been performed, and such comparisons have rarely been carried out in a blinded fashion. Another major issue inherent to longitudinal studies of atrophy is the lack of ground truth. In an attempt to address this, Fox et al. (2011) proposed desirable characteristics that atrophy measurement techniques should exhibit, including: symmetry, transitivity, comparisons with results from manual measurements, comparison with more established techniques (Schott et al., 2006), and reproducibility of measurements over short time intervals. For these purposes, we created the MIRIAD atrophy challenge, where we provided a common dataset of serial MRI data that could be used for a comparison of atrophy measurement techniques. We chose the Minimal Interval Resonance Imaging in Alzheimer's Disease (MIRIAD) dataset (Malone et al., 2013) consisting of 708 T1-volumetric scans acquired from 46 patients with a clinical diagnosis of AD and 23 controls scanned on multiple occasions over a variety of different intervals on the same 1.5 T scanner, and by the same radiographer; these data have since been made an open-access resource.^3^ Particular features of this dataset include the availability of short-interval follow­up scans, 2 and 6 weeks from the initial baseline scan, allowing for thorough investigation into repeatability of atrophy measurements; and a means of assessing within-day reproducibility as most subjects had 2 viable back‐to‐back scans at 3 of the time points. In previous studies, these scans have been used to perform atrophy simulation modelling (Camara et al., 2008) and to estimate sample sizes for clinical trials using both manual and automated techniques (reviewed in Malone et al., 2013), but aside from one paper comparing results between BSI and SIENA (Smith et al., 2007), little work has been done using this dataset to directly compare different techniques.

In designing the MIRIAD atrophy challenge we decided that an important component for comparing the different techniques of atrophy measurement would be the required sample sizes for clinical trials. The rationale for this choice is that given that other aspects of the design are fixed and the different methods provide reliable and repeatable measures across the cohort, there is clear utility in using methodology for measuring the outcome variable that requires the smallest sample size to provide 80% (sometimes 90%) statistical power to demonstrate a statistically significant treatment effect if the treatment under consideration truly has a clinically important disease-modifying effect (i.e., slowing the rate of atrophy compared to placebo). Numerous other factors can affect sample size, including the choice of outcome variable, the length of the trial, the number of interim visits, the anticipated number and pattern of dropouts and the method of statistical analysis. However, in this challenge these factors have been applied consistently across groups, so that the primary effect on sample size is the atrophy measurement technique itself.

In this paper, we describe the results from the MIRIAD atrophy challenge. From the submissions received from challenge participants, we obtained measures of short-term repeatability, symmetry, and transitivity (Fox et al., 2011), as well as estimates of the rates of atrophy for both AD and control groups in order to calculate required sample sizes for hypothetical clinical trials. These sample size estimates provide head-to-head comparisons of the effectiveness of these techniques.

## Materials and methods

### Data

Full details about the MIRIAD dataset, including how to obtain the data, can be found in Malone et al. (2013). In brief, the dataset consists of scans from 46 patients fulfilling National Institute of Neurological and Communicative Disorders and Stroke–Alzheimer's Disease and Related Disorders Association (NINCDS-ADRDA) working group criteria (McKhann et al., 1984) for probable AD and 23 age-matched, healthy elderly controls scanned multiple times over a follow-up period of up to 2 years, with subjects being scanned twice in one session at some visits. The basic demographics of the MIRIAD subjects can be found in Table 1. All scanning was done on a single 1.5-T scanner (GE Signa, GE Medical Systems, Milwaukee, Wisconsin) from 2000 to 2003. Volumetric T1-weighted images were acquired with an IR-FSPGR (inversion recovery prepared fast spoiled gradient recalled) sequence, field of view 24 cm, 256 × 256 matrix, 124 1.5 mm slices in coronal orientation, TR 15 ms, TE 5.4 ms, flip angle 15°, and TI 650 ms.

### MIRIAD atrophy challenge

The MIRIAD atrophy challenge was announced as part of the MICCAI 2012 Workshop on Novel Imaging Biomarkers in Alzheimer's Disease (NIBAD12) (Wang et al., 2012). Participants requested a login and then downloaded data from a dedicated XNAT (eXtensible Neuroimaging Archive Toolkit) (Marcus et al., 2007) server, which contained all the image data in NIFTI (Neuroimaging Informatics Technology Initiative) format. For each subject, only one of the baseline scans, henceforth referred to as the *identified baseline*, was identified to the participants. The rest of the temporal ordering was blinded to the participants. This blinding was performed to ensure that no added optimization could be performed when doing change measures on back-to-back or short interval scans. Participants submitted volumetric measures for every dataset in the challenge, as well as change measurements between each time point combination, both in the “forward” direction (e.g., baseline to 12 months) as well as in the “backward” direction (e.g., 12 months to baseline), so that symmetry could be tested. Volume and change measures were requested for the whole brain, ventricle, and both left and right hippocampi. The organizers checked the received data files with the participants to ensure that there were no problems during transfer and then unblinded the time point ordering for statistical analysis. The initial results of the MIRIAD atrophy challenge were presented in oral form as part of the NIBAD workshop. After the workshop, participants were allowed to submit outlier images that were not of suitable quality for their analysis methods. The organizers determined consensus outliers based on the feedback from all participants and image quality checks performed during the original data collection process. A consensus set of nine images from seven subjects (190_F, 217_G, 235_B, 237_E, 240_C, 255_A, 255_H, 256_A, 256_C), corresponding to 128 pair-wise measurements, was marked for removal from the analysis. All participants, whilst still blinded to timepoint and disease status, were given the option to re-run their analysis with the outliers excluded.

A total of nine groups participated in the challenge. Several groups submitted more than one method. Some groups only reported results on a subset of structures, and one group provided measurements only in terms of percentage change. An overview of each pipeline is presented in Table 2 and detailed results can be found in both the proceedings (Wang et al., 2012) and the Supplementary material.

### Reliability measurements

We measured three key aspects of atrophy measurement techniques as defined by Fox et al. (2011) that focus on plausibility and consistency. For all analyses, the data was grouped according to disease (healthy control and AD) in order to determine whether the behaviour of these measurements was different based on the population.

First, volumes of brain, lateral ventricles, left and right hippocampi from the identified baseline scans were compared between the two disease groups. The next assessment was to measure change over short interval scans. Specifically, the mean atrophy between the identified baseline and the other baseline scan, where available, was calculated. Two-tailed *t*-tests were performed on the null hypothesis that the mean change was zero. A similar analysis was performed using the identified baseline scan and the two-week scan. Whilst the variability at two weeks should be larger, the change would still be expected to be near zero. All measures of short interval change were standardized by the volume of the structure at the identified baseline scan, using the following formula for data when volumetric measurements were used:

(1) $$c_{\mathrm{indirect}}=100*\ln\frac{V_{r}}{V_{b}}$$

where *V_b_* is the volume measure at the identified baseline scan, and *V_r_* is the volume measure at the repeat scan. For methods where direct measures of change were available, the change was measured as:

(2) $$c_{\mathrm{direct}}=100*\ln\frac{V_{b}+\Delta V\left(b,r\right)}{V_{b}}$$

where ∆*V*(*b*,*r*) is the (forward) measure of direct change between the baseline and repeat scan. In the case of the INRIA group, where percentage change was the quantity provided, the formula used was *c_direct_* = 100 ∗ ln(1 + ∆*V*(*b*,*r*) / 100). All of the values were log (ln) transformed, as this gives a scale that approximates a percent change but is multiplicatively symmetric.

The final two measurements investigated the longitudinal consistency of the methods regarding symmetry and transitivity. These measures were only obtained for methods doing direct measures of atrophy since indirect measures are based purely on subtraction and thus are inherently symmetric and transitive. Two of the direct methods enforced symmetry in the algorithms: UCL-BSI by averaging the forward and (negative) backward atrophy measurements to obtain final values for both directions and INRIA by the symmetry of their registration algorithm. The symmetry difference, *d_sym_*, was calculated by looking at the ratio of the difference between the two measurements, and the average measurement of changes:

(3) $$d_{sym}=100*\frac{\Delta V\left(b,r12\right)-\Delta V\left(r12,b\right)}{0.5\left(\Delta V\left(b,r12\right)+\Delta V\left(r12,b\right)\right)}$$

where Δ*V*(*b*,*r*12) is the forward direct measure of volume change from the identified baseline to the repeat scan at month 12, and Δ*V*(*r*12,*b*) is the negative of the backward direct measure of volume change (which will itself usually be negative since brain volume is typically lost over time) from the repeat month 12 to the identified baseline. For transitivity, the difference was measured as

(4) $$d_{\mathrm{trans}}=100*\frac{\Delta V\left(b,r12\right)-\left(\Delta V\left(b,r6\right)+\Delta V\left(r6,r12\right)\right)}{0.5\left(\Delta V\left(b,r12\right)+\left(\Delta V\left(b,r6\right)+\Delta V\left(r6,r12\right)\right)\right)}\text{.}$$

In some cases, where the amount of change in the actual subject was quite small (most of the controls), differences very small in magnitude could result in large errors and outliers. As a result, these differences were reported using median (with 95% confidence intervals) rather than the mean.

### Statistical analysis of imaging outcome measures

A number of different statistical approaches can be used to analyse a clinical trial with an imaging outcome, with choice between them in part dependent upon the complexity of the design. With only a single *direct* (Frost et al., 2004) measure of change (such as a measure of atrophy derived from a boundary shift integral (Freeborough and Fox, 1997)) a two-sample *t*-test can be used (provided normality assumptions hold and/or the sample size is large). Another option is to measure brain volume at baseline (pre-randomization) and at the end of follow-up, producing an *indirect* measure of change as calculated by subtracting the two volumes, with a *t*-test again being used to compare the treatment and placebo groups. Alternatively, the baseline can be used as a covariate in an analysis of covariance (ANCOVA), an approach that increases statistical power (Frison and Pocock, 1992). With multiple repeated measures, a variety of approaches are possible, with linear mixed models one option (see Frost et al., 2008 for sample size requirements when using linear mixed models). However, for simplicity in the analysis presented here, we restrict attention to designs where patients are seen only once pre-, and once post-, randomization. For designs where the outcome is an *indirect* measure of change (calculated by subtraction of two volume measures) we assume that the trial will be analysed using a comparison of changes, rather than using ANCOVA. Here we consider clinical trials of length 6, 12 and 24 months, three intervals over which trials of disease-modifying therapies are often considered.

To carry out a sample size calculation for a particular clinical trial design with an imaging outcome, two quantities are key. First, it is necessary to specify the anticipated treatment effect on the mean atrophy rate. The current consensus is that a 25% slowing of atrophy would likely be a clinically significant disease modification. Whether this 25% slowing should be in relation to the atrophy rate seen in a healthy ageing population or an absolute slowing is still an ongoing discussion in the community (Holland et al., 2012, Hua et al., 2013). Here, we adopt the former approach.

The second key quantity is the anticipated variance of the estimated treatment effect. For the simple trial designs considered here where the outcome is a measure of rate of change over a single time-interval, this variance is a simple function of the sample size and the variance of the rate of change. This variance is expected to decrease with increasing follow-up time, since the within-subject component will decrease over time until the overall variance asymptotically approaches the between-subject variance (Schott et al., 2006). With repeated measures over multiple time-points variances of changes over particular time intervals can be separately empirically estimated. However this approach can give rise to implausible behaviour through the play of chance, with for example variances first increasing and then decreasing with increasing follow-up. Our preferred approach and the one adopted here is to first identify a linear mixed model (Verbeke and Molenberghs, 2000) that fits the data well and then to use the estimated parameters from this model to predict variances of changes over all the time intervals of interest and hence required sample sizes for clinical trials. To do this we make the commonly adopted assumption that variability will be unaltered by treatment and that variability in both groups will mimic that seen in the observational cohort of patients.

### Model for analysis of repeated measurements of brain volume

The model for repeated observations on subjects in a single group is a random slope model with fixed subject effects. Since the MIRIAD data includes measures from more than one scan at some visits the model also incorporates random visit effects.

(5) $$\begin{array}{l} y_{ijk}=\alpha_{i}+\left(\beta \;+\;b_{i}\right)t_{ij}\;+d_{ij}+e_{ijk}\\ \mathrm{with}\;b_{i}\sim N\left(0,\sigma_{b}^{2}\right),\;d_{ij}\sim N\left(0,\sigma_{d}^{2}\right)\;\mathrm{and}\;e_{ij}\sim N\left(0,\sigma_{e}^{2}\right) \end{array}$$

Here *y*_*ijk*_ is the value of the outcome variable (e.g., brain volume) for the *i*th subject, attending their *j*th visit (*j* = 1 to 9) with the measurement made at the *k*th scan (*k* = 1 at most visits, but 1 or 2 at those visits with repeat scans) and *t_ij_* is the time of the *j*th visit (in years) relative to the baseline visit. The *β* term is the fixed effect representing the mean atrophy rate per unit time, whilst *b_i_* is the subject-level random effect that allows for variability in atrophy rate between subjects. The terms *d_ij_* and *e_ijk_* together represent within-subject variability over and above that accounted for by atrophy for the *i*th subject. This is partitioned into between-visit (*d_ij_*) and within-visit between-scan variability (*e_ijk_*). From the model in Eq. (5) the difference between measures at visit *j*_1_ (scan *k*_1_) and at visit *j*_2_ (scan *k*_2_) for the *i*th subject is given by the following formula.

(6) $$y_{ij_{2}k_{2}}-y_{ij_{1}k_{1}}=\left(\beta \;+\;b_{i}\right)\left(t_{ij_{2}}\;-t_{ij_{1}}\right)\;-\;d_{ij_{1}}+d_{ij_{2}}\;-\;e_{ij_{1}k_{1}}+e_{ij_{2}k_{2}}$$

Using the general results that Var(*A* + *B*) = Var(*A*) + Var(*B*) provided that *A* and *B* are independent and that Var(*cA*) = *c*^2^(Var(*A*)) where *c* is a constant, the implied variance of this difference is given by the following formula.

(7) $$Var\left(y_{ij_{2}k_{2}}-y_{ij_{1}k_{1}}\right)\;=\;{\left(t_{ij_{2}}-t_{ij_{1}}\right)}^{2}\sigma_{b}^{2}+2\sigma_{d}^{2}+2\sigma_{e}^{2}$$

It follows that the implied variance of a rate of change derived from a difference between two measures on the same subject is as follows.

(8) $$Var\left[\left(y_{ij_{2}k_{2}}-y_{ij_{1}k_{1}}\right)\;/\left(t_{ij_{2}}-t_{ij_{1}}\right)\right]=\sigma_{b}^{2}+2\left(\sigma_{d}^{2}+\sigma_{e}^{2}\right)/{\left(t_{ij_{2}}-t_{ij_{1}}\right)}^{2}$$

In Eq. (8) *σ*_*b*_^2^ is the between-subject variance in rates of change whilst the remainder of the variance formula represents the within-subject variance. Provided that the model is correct, the latter decreases with increasing follow-up whilst the former remains constant, as mentioned in the Statistical Analysis of Imaging Outcome Measures.

### Model for analysis of repeated “direct” measures of change in brain volume

Here the model for repeated measures on subjects in a single group extends that recommended by Frost et al. (2004) for repeated “direct” measures of change. Again the basic model is extended to incorporate random visit effects as follows.

(9) $$\begin{array}{l} c_{ij_{1}k_{1}j_{2}k_{2}}=\left(\beta \;+\;b_{i}\right)\left(t_{ij_{2}}\;-t_{ij_{1}}\right)\;-\;u_{ij_{1}}+u_{ij_{2}}\;-\;v_{ij_{1}k_{1}}+v_{ij_{2}k_{2}}+w_{ij_{1}k_{1}j_{2}k_{2}}\\ b_{i}\sim N\left(0,\sigma_{b}^{2}\right),\;u_{ij}\sim N\left(0,\sigma_{u}^{2}\right),\;v_{ijk}\sim N\left(0,\sigma_{v}^{2}\right),\;w_{ij_{1}k_{1}j_{2}k_{2}}\sim N\left(0,\sigma_{w}^{2}\right) \end{array}$$

Here $c_{ij_{1}k_{1}j_{2}k_{2}}$ is the measured change between visit *j*_1_ (scan *k*_1_) and visit *j*_2_ (scan *k*_2_) for the *i*th subject. The *u*_*ij*_s are subject-specific random visit effects impacting on any “direct” change measured from, or ending at, the *j*th visit. Two such visit effects impact on each scan pair, with that for the “start” visit having a negative sign and that for the “end” visit having a positive sign. The *v*_*ijk*_s are analogous effects relating to scans, whilst $w_{ij_{1}k_{1}j_{2}k_{2}}$ is unexplained residual variability.

The model here has strong parallels with that for direct differences (Eq. (6)). These parallels, and the rationale for these models, are discussed in detail in Frost et al. (2004). In brief the use of direct measurements is expected to reduce the variance of the visit and scan effects (i.e., *σ*_*u*_^2^ and *σ*_*v*_^2^ in Eq. (9) are expected to be markedly smaller than *σ*_*d*_^2^ and *σ*_*e*_^2^ in Eq. (6) respectively) whilst the $w_{ij_{1}k_{1}j_{2}k_{2}}$ terms in Eq. (9), which arise because direct measures of change are not perfectly additive, are typically small in magnitude.

For the model specified in Eq. (9) the implied variance of a measure of change is as follows.

(10) $$Var\left(c_{ij_{1}k_{1}j_{2}k_{2}}\right)=\;{\left(t_{ij_{2}}-t_{ij_{1}}\right)}^{2}\sigma_{b}^{2}+2\sigma_{u}^{2}+2\sigma_{v}^{2}+\sigma_{w}^{2}$$

It follows that the implied variance of a rate of change is as follows.

(11) $$Var\left[\left(c_{ij_{1}k_{1}j_{2}k_{2}}\right)/\left(t_{ij_{2}}-t_{ij_{1}}\right)\right]=\sigma_{b}^{2}+\left(2\sigma_{u}^{2}+2\sigma_{v}^{2}+\sigma_{w}^{2}\right)/{\left(t_{ij_{2}}-t_{ij_{1}}\right)}^{2}$$

As with Eq. (8) here *σ*_*b*_^2^ is the between-subject variance in rates of change whilst the remainder of the variance formula represents the within-subject variance, which decreases with increasing follow-up.

### Required sample sizes for clinical trials

Assuming that a putative treatment can reduce the excess atrophy rate (as discussed above, for the purposes of this study over and above that seen in healthy controls) by 25% without altering variability, standard formulae give the following sample size requirements for a trial with a single measure of change and equal numbers in each group, if that trial is to have 80% statistical power to detect a treatment effect using a conventional two-sided significance level of 5%.

(12) $$N=2\times {\left[\left(1.960+0.842\right)/\left(0.25\times ES\right)\right]}^{2}$$

(13) $$\mathrm{where}\;ES=\left(\beta_{\mathrm{Case}}-\beta_{\mathrm{Control}}\right)/\sqrt{Var\left(\mathrm{rat}e_{\mathrm{Case}}\right)}$$

Here *N* is the required sample size per group, *β*_*Case*_ and *β*_*Control*_ are the respective mean rates of change and *Var*(*rate*_*Case*_) is the relevant variance of the rate of change (Eq. (8) or Eq. (11)) in patients with Alzheimer's disease (cases). *ES* is an effect size: a standardized measure of the difference in the mean rate of atrophy between two groups. This formula can be used for trials with a single “indirect” or “direct” measure of change for each subject. In the former case the approach is mathematically equivalent to that proposed by Diggle et al. (2002).

### Transformations, omitted points and other modelling considerations

Whole brain atrophy and hippocampal atrophy are typically expressed as percentage changes and hence most appropriately analysed after logarithmic transformation. Frost et al. (2004) provide details of the exact methodology. Ventricular expansion rates are most commonly analysed in absolute terms of volume change without such a transformation or normalization to baseline volume. However, we found that absolute rates of change in this cohort appeared to increase with increasing follow-up, implying that a percentage change for ventricle expansion was more appropriate. Thus, and for consistency with the other outcomes, we utilized a logarithmic transformation for all measures.

### Confidence intervals for sample sizes and head-to-head comparisons

When attempting to compare imaging techniques on the basis of sample size estimates it is important to consider uncertainty in sample size calculations. We did this using bootstrap resampling methodology (Efron and Tibshirani, 1994). Specifically we constructed non-parametric, bias-corrected and accelerated (BCa) confidence intervals from 2000 bootstrap samples for each of our sample size estimates. Each bootstrap sample included the same number of AD cases and controls as the original sample. The confidence intervals were constructed on the “effect size” scale, since the distribution of estimated effect sizes is likely to be more symmetric than that of estimated sample sizes and so confidence intervals calculated on this scale are likely to have better coverage properties. Bootstrap confidence intervals were also calculated for the between and within components of variance for the linear mixed models using an analogous approach.

Head-to-head comparisons of sample size requirements between pairs of imaging methodologies were made by considering the distribution of the paired differences in effect sizes estimates over the 2000 bootstrap samples. Where more than 97.5% of these comparisons were in favour of one of the methodologies the difference was judged statistically significant at the 5% level (two-sided test).

For most techniques and most structures the models specified in Eqs. (5), (9) converged, yielding reliable parameter estimates when fitted in Stata 12. However on occasions convergence problems were encountered either when fitting the model to the full data or to bootstrap samples drawn from the data. This occurred when one of the components of variance was estimated to be zero or close to zero. For six structure–technique combinations (BAUMIP for left and right hippocampi, CSIRO for left hippocampus, INRIA for right hippocampus, MAYO_BSI for whole brain and MNI for right hippocampus) such convergence problems were encountered in more than 1% of the bootstrap resamples. For these structure–technique combinations the relevant model in controls was replaced by one constraining both *σ*_*b*_^2^ and *σ*_*d*_^2^ (Eq. (5)) or both *σ*_*b*_^2^ and *σ*_*u*_^2^ (Eq. (9)) to be 0. For all but the BAUMIP-left hippocampus combination (where convergence was not achieved in 2.35% of bootstrap samples) this modification to the model reduced the number of samples with convergence problems to below 1%. The impact of this modification to the model for controls had a negligible impact on the required sample size in each setting.

## Results

The Supplemental Table shows the extent of all of the data collected in the MIRIAD study and previously reported, highlighting the aforementioned 9 scans and 128 corresponding image pairs from 7 subjects that were excluded here on quality grounds. Subjects were seen at baseline, 2, 6, 12, 26, 38, 52, 78 and 104 weeks with repeat scans taken on three occasions (baseline, 6 and 38 weeks). All 23 controls and 44 out of the 46 AD cases attended the 52-week follow-up with high retention rates at the visits in the intervening period. A subset of the subjects returned for additional scans at 78 and 104 weeks that were not part of the original imaging protocol. Each subject provided measures of “direct” change for up to sixty-six (12 × 11/2) scan pairs with three of these being same day scan pairs.

**Fig. 1:**
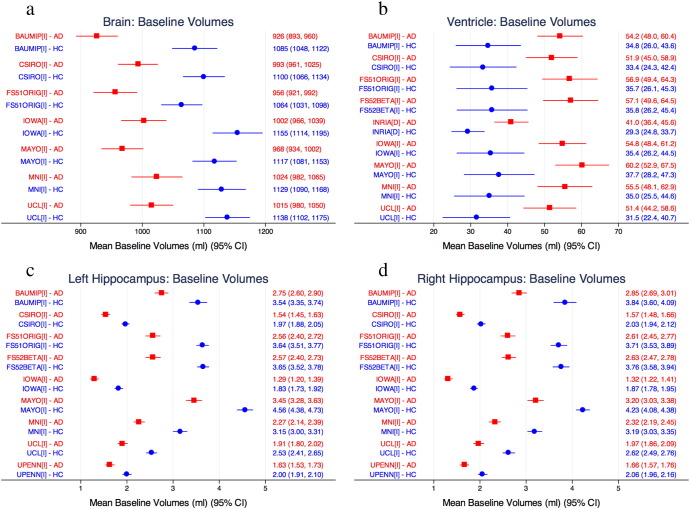
Baseline volumes of each method for (a) whole brain, (b) lateral ventricles, (c) left hippocampus and (d) right hippocampus for all groups. Red squares indicate the AD patient group and blue circles indicate the control group for each technique.

Fig. 1 shows the mean and 95% confidence interval of the raw volume measurements (uncorrected for overall head size) on the identified baseline scan, separated by disease group, for all four structures and for each technique. There was good group separation across all structures between AD and controls (no pair of 95% confidence intervals overlapped) for each technique. The ventricles were overall the most consistently measured. The whole brain had more variability, but was more consistent compared to the hippocampi, where there was a 2.3- to 2.7-fold difference in volume between the techniques that had the smallest mean hippocampal volume and those with the largest.

**Fig. 2:**
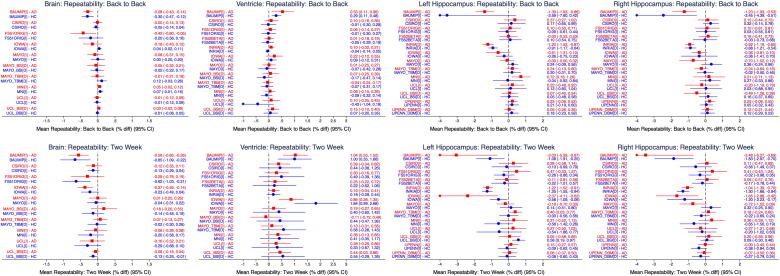
Back to back and two week repeatability measures for all four regions. All measures are provided in terms of % difference from baseline. Red squares = AD, blue circles = controls. Diamond markers represent truncation of the confidence interval if out of range.

Fig. 2 shows the short-term repeatability measures for all four structures, with the left column representing the same day measurement and the right column representing the two week change. Whilst most of the methods have 95% confidence intervals spanning 0, providing no statistically significant evidence of change, methods from three groups did provide some evidence of bias. The Iowa group had atrophy rates that were significantly different from zero in the two-week repeatability measure for all four regions for the AD group and three of the four (ventricle and hippocampi) for the control group. The INRIA group had non-zero atrophy rates for the hippocampi in all groups, both for the back to back and two week measures. Finally, all but one of the BAUMIP measures of mean short-term atrophy were significantly different from 0.

Table 3, Table 4 provide results from the symmetry and transitivity assessments for the direct change methods. Most methods show very small differences in these measures, with the main exception being the hippocampi of the controls.

**Fig. 3:**
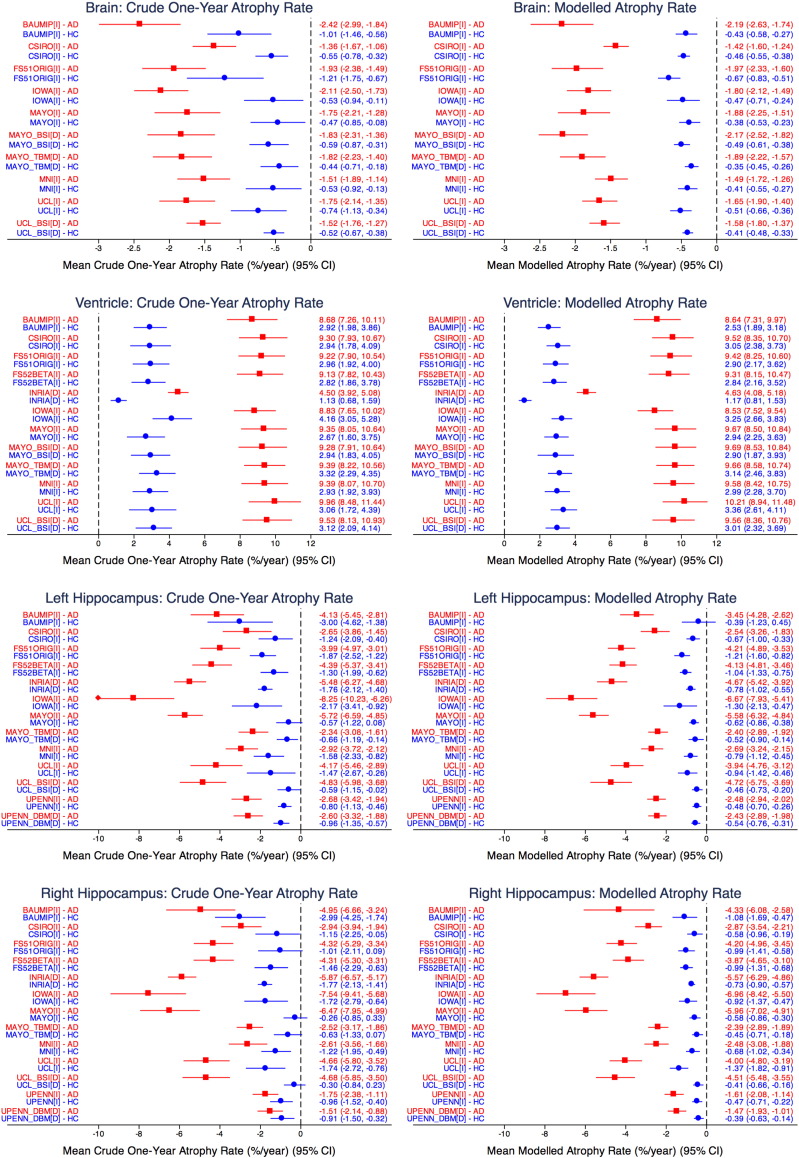
Mean atrophy rates with 95% confidence intervals estimated (left) from a single pair of scans 12 months apart and (right) from all available data using statistical linear mixed models.

Fig. 3 shows mean atrophy rates with 95% confidence intervals estimated both from single pairs of scans 12 months apart and from all available data using our linear mixed model approach. Results are shown for each technique and each structure, separately for AD cases and healthy controls. Typically the differences between the techniques are similar whether the one-year rates or the modelled rates are considered, but confidence intervals are markedly reduced when considering results that utilize all of the data. Modelled estimates of the mean whole brain atrophy rate in AD subjects differ according to the technique used – in some cases showing significant differences between techniques – from a minimum of 1.42%/year (95% CI 1.24 to 1.60%/year) to a maximum of 2.19%/year (95% CI 1.74 to 2.63%/year). Mean whole brain atrophy rates in controls are more homogeneous across techniques, as are ventricular atrophy rates in both groups. In contrast hippocampal atrophy rates, particularly in AD subjects, differ quite markedly across techniques. Here 95% confidence intervals for the means frequently do not overlap.

**Fig. 4:**
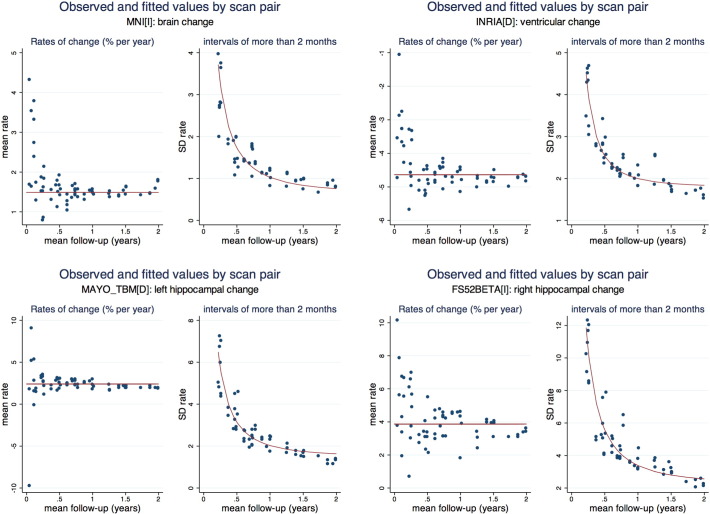
An illustration of the extent to which the statistical models fit the data, using one technique for each structure and selected techniques as exemplars. For each of the sixty-three scan pairs (sixty-six less the three same-day scan pairs) the empirical mean rates are contrasted with those predicted by the linear mixed model.

Fig. 4 illustrates the extent to which the models fit the data, using one technique for each structure as exemplars. For each of the sixty three scan pairs (sixty-six less the three same-day scan pairs) the empirical mean rates are contrasted with those predicted by the linear mixed model. The figures illustrate the extent to which means and standard deviations can differ when estimated empirically even when follow-up times are the same or similar. For each of the techniques shown, mean rates appear approximately constant, at least over time intervals of more than a few weeks. In addition standard deviations of rates of change decrease as follow-up increases, with the pattern of the decline well described by the linear mixed model. In general similarly good fits of the models to the data were seen for the other techniques.

Table 5 presents the between (*σ*_*b*_^2^) and within (*σ*_*w*_^2^) components of variance in atrophy rates for each technique and each structure in the AD patients. These can be used to compute the total variance over any follow-up time of *t* years (*σ*_*b*_^2^ + *σ*_*w*_^2^/*t*^2^). The formula indicates that – as expected, and previously demonstrated (Schott et al., 2006) – the longer the follow-up the greater the importance of the between subject variance.

Table 6 presents the required total sample sizes (both groups combined) for 6, 12 and 24 month clinical trials for each structure and for each technique. 95% bootstrap confidence intervals are also shown. Results shown in bold and underlined purple text are those requiring the smallest sample size for each structure and time interval. Those shown in underlined and green text are not statistically significantly worse than those shown in purple, as assessed through pairwise comparison of the bootstrap sample size estimates.

For whole brain atrophy the UCL boundary shift integral approach gives the smallest required sample sizes. For six month trials the required sample size with this technique is statistically significantly smaller than those for all other techniques. For two year trials the UCL technique gives the smallest sample size, but the advantage over several of the other techniques is not statistically significant. This reflects the fact (seen in Table 2) that the UCL technique has markedly smaller within-subject, but not between-subject variability than the other techniques.

**Fig. 5:**
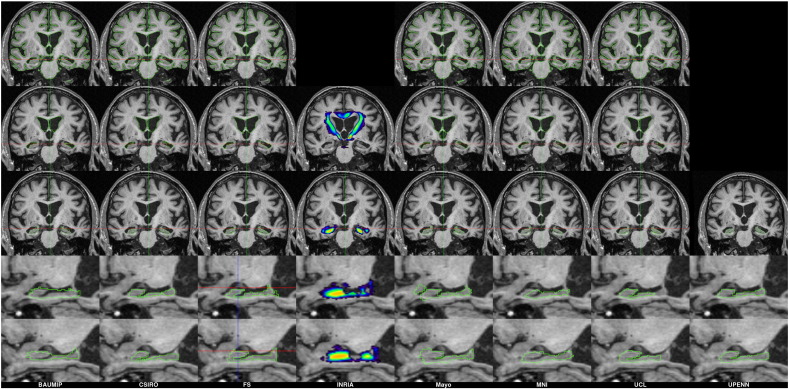
Sample region delineation from MIRIAD atrophy challenge subject 220A. Each column represents a submission and the rows show a different region outlined in green (from top to bottom): whole brain, lateral ventricles, and hippocampi. In one case, INRIA, only a probabilistic mask was used, and this is shown with colour overlays.

For ventricular expansion, there were many submissions (8 submissions at 6 months, and 3 submissions at 12 and 24 months) that show no statistically significant evidence that they are worse than the lowest sample size achieved by the INRIA technique. INRIA's rates of ventricular expansion, and the associated variances, are much lower than the rest of the groups. It is worth noting that INRIA's definition for the region over which ventricular expansion is computed not on a traditional anatomical basis (see Fig. 5), but rather through identifying the most sensitive areas of contraction and expansion estimated through longitudinal non-linear registration in an independent training set (ADNI). For this reason, the resulting probabilistic region is determined at the same time by the anatomical information represented in the images, and by the underlying registration model (LCC-logDemons). In particular, the localization of the sensitive areas of volume changes reflects the smoothness assumptions of the registration algorithm. As with the other structures considered here marked reductions in sample size are achieved by extending follow-up from 6 to 12 months, with the benefit of further extension to 24 months less marked.

For hippocampal atrophy there are very marked differences in sample sizes according to the technique used. For the right hippocampus, required sample sizes are smallest using the INRIA technique with all comparisons with other techniques statistically significant. For the left hippocampus the INRIA technique gives the smallest required sample size over 6 months, but this is very similar to that for the Mayo technique, which becomes the most efficient over longer follow-up intervals. Whilst the sample sizes are the lowest, there is evidence from the short-term repeatability measures that INRIA's hippocampal measures has evidence for some bias in both the AD and control groups.

## Discussion

We have performed an extensive statistical analysis making comparisons among what we believe to be the largest collection of different imaging techniques ever compared head-to-head in a blinded single analysis. The comparative analysis has been carried out using the MIRIAD dataset, which although not the largest data-set in terms of numbers of subjects, has the advantage of including multiple measures of atrophy over a range of different intervals from two weeks up to two years. This makes the MIRIAD dataset particularly useful for accurately quantifying the between and within subject variability in rates of atrophy; this variability is a key component of required sample sizes for hypothetical clinical trials in AD, our primary outcome measure.

All participants provided measurements for every dataset in the challenge, allowing for a more accurate comparison between methods. In an effort to encourage researchers to provide clearer comparisons between different methods and research studies on the most widely used cohort in the community, the Alzheimer's Disease Neuroimaging Initiative (ADNI) has created standardized analysis datasets (Wyman et al., 2012). The fact that all techniques were assessed on exactly the same data, with only minimal exclusion of poor quality scans is a strength of our analysis. In other comparative studies, the number of images included for each technique has not always been controlled. For example, in the analysis reported by Holland et al. (2012), the number of subjects included in the analysis ranged from 572 to 733 depending on the method. As well as being consistent across techniques we felt that, since our aim was to compute required sample sizes for hypothetical treatment trials, the number of exclusions should be small. This is because an important consideration in assessing the reliability of results from any clinical trial is the extent of missing data since this has the potential to introduce bias. For example, a study by Hua et al. (2013) illustrated that selective data exclusion, albeit based solely on post-hoc removal of data with implausible atrophy rates, will result in a bias towards lower required sample sizes.

A further strength of our analysis is our use of linear mixed effect models. The MIRIAD data has been used in a similar manner before (Smith et al., 2007) to compare BSI, SIENA, and SIENAX. In their study, they also obtained measures of repeatability using the same day scan and transitivity. However, instead of using linear mixed effect models, the comparison of atrophy rates was done by comparing *t*-statistics on whole brain atrophy measures of the first and last scans for each subject. The incorporation of linear mixed effect models in the MIRIAD atrophy challenge allows for estimates of the various components of variance that determine required sample sizes. Variances could alternatively have been separately empirically estimated for each interval. The advantage of using a linear mixed model is that implausible inconsistencies (such as variances changing non-monotonically with increasing scan interval) are eliminated with variance components estimated in a statistically efficient manner using all available data. The linear mixed model used for the repeated “direct” measures of change (Frost et al., 2004) is less simplistic than that used by others (Bernal-Rusiel et al., 2012, Holland et al., 2012) in that it incorporates visit (and scan effects) which act in different directions depending upon whether a particular “direct” measure starts from, or ends at, the visit (scan) in question. These visit and scan effects, which were statistically significant in almost all models, should in our opinion be routinely included in all analysis models for repeated measures of “direct” change.

The structure that was most consistent across all groups was the lateral ventricles, with sample sizes for a one year trial of the order of 200 to 300 for most techniques. This is likely due to the fact that the boundary around this structure is clear and distinct, primarily CSF bordered by white matter, and the anatomical definition is consistent across centres. A previous study (Kempton et al., 2011) validated different lateral ventricle segmentation algorithms (ALVIN, FSL FIRST, FreeSurfer) using numerous criteria, including similarity to a “gold standard” manual labelling, reproducibility between different sequences and scans with intervals of 90 days or less, and sensitivity to longitudinal change. The results also showed high agreement within the AD and elderly population between all methods. Whilst changes in the ventricles are sensitive measures of brain atrophy, as a large percentage of tissue loss is reflected in ventricle expansion, it is not a very specific measure in terms of dementia, as there can be numerous reasons for ventricle expansion and contraction besides atrophy, including hydration. Different subtypes of dementia will also show similar rates of ventricle expansion.

Whole brain measurements, whilst not as consistent as the lateral ventricles, still showed a reasonable level of consistency across groups. Required sample sizes were typically larger than for ventricular atrophy. The drop in consistency compared to lateral ventricles is likely due primarily to partial volume effects, which will be much greater on the convoluted cortical surface when compared to the more simple structure of the ventricle. In validation studies on different brain segmentation algorithms (Eskildsen et al., 2012, Leung et al., 2011a), voxels on the cortical surface boundary, especially those near the temporal lobe, seemed to be the most likely source of mis-segmentation.

In both whole brain and ventricles, resulting sample size estimates correspond well with the sample sizes estimated from the ADNI cohort using the same sample size formula and atrophy measurement methods. For example, the estimated sample size for a one year study using the UCL KN-BSI method in the brain region was 242 (152 to 422) in this study and 220 (148 to 353) for ADNI (Leung et al., 2010b). This was replicated by Holland et al. (2012) who used the KN-BSI data uploaded to ADNI from UCL and obtained a two year sample size estimate of 180 (129 to 276) for two years; in this cohort, the estimate was 188 (114 to 336). For ventricles, the sample size estimate is 190 (118 to 320) for MIRIAD and 257 (177 to 417) from ADNI. The slightly larger differences may be that Holland and colleagues did not have access to the KN-BSI results in ADNI for the ventricles. Holland et al. study also provided sample size estimates for FreeSurfer using the ADNI data, though this might have been performed on a different software version compared to the challenge. For whole brain, the estimate for ADNI was 696 (393 to 1693) and for MIRIAD 432 (220 to 1108) and 646 (276 to 7832) for the two submissions. For ventricles, the values were 255 (174 to 427) for ADNI and 186 (112 and 312) and 182 (112 to 314) for the challenge. More formal comparisons of these sample size estimates across data cohorts will be of interest and the focus of future work.

The least consistent structure across all assessments was the hippocampi, with required sample sizes sometimes varying as much as ten-fold between techniques. This variability between techniques is likely due to the numerous definitions of the hippocampus used in segmentation protocols. Fig. 5 provides example delineations of the same image from all the challenge techniques for a single subject. This discrepancy has lead to an EADC initiative (Boccardi et al., 2011, Frisoni et al., 2015) to create a unified hippocampal segmentation protocol based on previous protocols from the literature and additional input from numerous experts during iterations of the universal protocol. Despite this variability, the results are in-line with a previous study using manual delineations of hippocampi in 36 patients and 20 controls from this dataset (Barnes et al., 2008), which reported 12 m rates of atrophy to be in the order of 4.5%/year for patients, and 0.3%/year for controls. A future comparison of hippocampal atrophy techniques based on a consistent ROI definition across participants could be of considerable interest.

As the hippocampus, and the temporal lobe in general, is one of the areas most likely to be affected by MRI acquisition artefacts (susceptibility, flow, motion), this could be another source of error in segmentation on other atrophy measurements. Since the hippocampus is such a small structure (roughly 0.2 to 0.4% of total brain volume), and images are discrete in nature, any mis-classification has a much greater effect on the results, with methods that compute change based on volumetric differences likely more affected than those based on direct change.

The techniques providing the sample sizes using hippocampal atrophy were similar in magnitude to those for ventricular atrophy. In general, compared to sample sizes reported using the ADNI dataset, hippocampal sample sizes are larger in MIRIAD. Using the UCL BSI technique in the hippocampi, sample size estimates were 490 (290 to 794) in MIRIAD and 135 (79 to 301) for ADNI (Leung et al., 2010a); and for FreeSurfer 284 (158 to 698) for MIRIAD and 217 (150 to 355) in ADNI (Holland et al., 2012). These higher sample sizes could be due to coarser resolution of the T1 weighted images in MIRIAD, more modern scanners and coils available in many of the ADNI sites or underlying differences in the AD population between the two cohorts.

For all analysis regarding required sample sizes, it is important to consider uncertainty in these estimates, as the point estimate may provide a very low sample size, but the resulting confidence intervals may be very wide. It is also important to directly compare sample sizes using significance tests, not rely on inferences made from the extent to which confidence intervals overlap. We use the non-parametric bootstrap to construct confidence intervals and make direct comparisons between sample sizes.

Most methods showed no clear signs of bias as indicated by their mean same day and two week atrophy rates. However there were a couple of exceptions (Baumip, INRIA, and Iowa). It is possible that these methodologies exhibit a systematic bias that is consistent across all groups, hence not affecting the sample size estimation, although this is not guaranteed. Measures of reliability and reproducibility are important factors to consider in addition to the sample size estimates when determining which imaging biomarker is most appropriate for use as an endpoint in a clinical trial. In the BAUMIP submission, the non-zero rates were almost certainly caused by the constraint that was placed in the pipeline where follow-up scans that were greater than the identified baseline (or in the case of the ventricles, less than the identified baseline) were not allowed to have a change of greater than 0. This constraint was based on the assumption that individuals with pathologies like gliomatosis cerebri that can affect the volume of brain parenchyma would be detected and excluded from any clinical study of AD. Whilst it is plausible to assume elderly subjects, whether they are healthy or have dementia, will not demonstrate brain growth, there are many sources of measurement error that might cause this effect within an MRI scan; and as demonstrated in previous therapeutic studies, brain swelling, perhaps reflecting therapy-related inflammation, can occur. The variability of these short interval atrophy rates also provides some insight on the repeatability of these measures. In this case, the spread of these errors was greater for the ventricle rather than the brain. As mentioned before, this could be due to the non-specific nature of ventricle change. The hippocampus again had the largest spread of errors. In another test–retest variability study, 5 subjects were recruited at 8 participating sites with different 3 T scanners, and two T1 scans were acquired 7 to 60 days apart. Morphometric results were computed using the cross-sectional and longitudinal FreeSurfer stream (Jovicich et al., 2014). The mean test–retest variability across all 8 sites was 1.8 ± 0.4 for hippocampus and 2.3 ± 0.4% for lateral ventricles. These test–retest variability values are similar to many of the participant's results in this challenge.

**Fig. 6:**
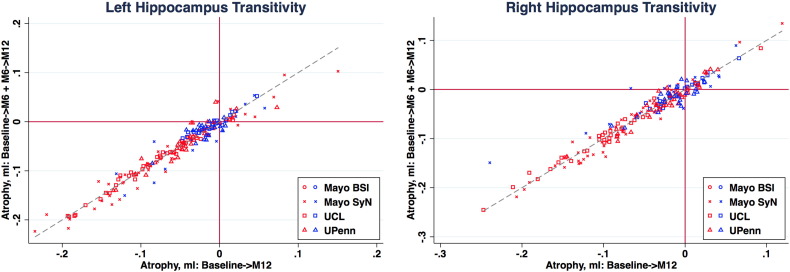
Transitivity plots comparing 12 month atrophy measures in the hippocampi. Blue points indicate controls and red points AD patients. Different point glyphs are used to distinguish methods. A dashed line at y = x is present to indicate where a perfectly transitive measure would be located.

For techniques that produced so-called direct measurements of change, symmetry and transitivity differences were assessed. In the brain and ventricle, these differences were small. In the hippocampus, particularly within the controls, these differences were very large when compared to the overall measured change. The UPENN_DBM method has median values that are elevated in the right hippocampi for AD compared to other groups, though the 95% confidence intervals still span across 0, indicating that there is likely no bias present. The larger values are likely due to there being very little actual change in the structure, thus it is a measurement with low signal and high noise. Fig. 6 provides plots of the hippocampi using the summed 6-month measurements on the vertical axis and the change using only the baseline and 12 month in the horizontal axis. For all the different methods, the controls are tightly centred around 0, so it is very possible that very small differences will represent a large percentage of the average amount of change. Additional measures of transitivity, including different normalization strategies, should be explored.

In all but two of the 12 cases listed in Table 3, the method producing the lowest sample size was a “direct” technique measuring change between two scan pairs. In the two cases where an indirect technique produced the lowest sample size (Mayo), there was no significant difference with a direct method (INRIA). Direct measures of change are desirable because they combine information from the two images together and measure the difference between these images, which should reduce the variability caused by segmentation errors. However, it is worth noting that many methods that are classified as indirect in this study actually incorporate information from all/other time-points within a subject to be more longitudinally consistent, which should reduce within subject variance, and produce lower sample size estimates than if data from fewer time-points was available. When incorporating information from multiple time-points to reduce variability, it is important to design these methods such that they ensure as little bias and loss of sensitivity as possible. Methods that incorporate longitudinal smoothing as a constraint have the potential of reducing sensitivity of actual changes between two individual time points. Since these indirect measures that incorporate longitudinal consistency typically improve with more data and the sample sizes are based upon using all of the data available for a subject, it is possible that our predictions for sample sizes for clinical trials involving just one pre- and one post-randomization measures are all underestimates of what would be required. However this does not invalidate using the approach that we have adopted for making comparisons between techniques.

Our analysis does have some other limitations. Due to space constraints we do not consider the effects of missing data on required sample sizes. Missing data can arise through subject drop out or by images not being deemed suitable due to poor quality. Its effects can be allowed for using a pattern-mixture approach as advocated by Dawson and Lagakos, 1991, Dawson and Lagakos, 1993 and described by Frost et al. (2008). We also here consider only simple trial designs with two visits, one at baseline and the other at the end of follow-up, ignoring the potential advantages of including interim visits in the design. Both of these issues are explored using the original MIRIAD data in the work of Schott et al. (2006). The method of blinding implemented in this challenge could be considered an obstacle in terms of getting the largest effective size. In terms of a tradeoff between bias and variability, we erred more on the side of removing potential sources of positive bias by blinding the participants to disease group, as well as the time between scans. As a result, it was more difficult for the participants to identify potential outliers and take corrective action. Many of these methods have been developed and validated using the ADNI datasets, acquired at both 1.5 T and 3 T, whereas all of the data from the MIRIAD atrophy challenge was acquired on a 1.5 T scanner using different parameters than those used in ADNI. Whilst most methods show good agreement whether the data was acquired on 1.5 T or 3 T scanners, such that they are often pooled for analysis in clinical trials, it is possible that the difference in protocols resulted in slightly different behaviour than that seen in ADNI. Finally, this data represents a snapshot of the state of the art techniques from those research laboratories who participated at the time when the MIRIAD atrophy challenge was completed. However, since that time, many of the participants have techniques that have continued to evolve and improve.

## Conclusion

In this paper, we provide a systematic framework utilizing a common data-set allowing a variety of techniques designed to measure change in brain, ventricles and hippocampi to be evaluated and compared. Despite the differences in techniques, challenge participants from around the world produced consistent and repeatable measures of change, particularly for the ventricle and the brain. Hippocampal measures are more variable, likely due to the differing definitions of the structure. We demonstrate that, in general, direct measures of change are associated with smaller variances than indirect measures; and that the statistical model previously designed to analyse multiple time-point whole brain atrophy is also able to model rates of hippocampal atrophy and ventricular expansion accurately, and thus to provide estimates of within and between subject variability in rates of change. Our results suggest that sample size estimates based on ventricular expansion rates are more consistent than those from whole brain atrophy, and both are markedly more stable than those derived from hippocampal atrophy measures. By providing comparisons between techniques based on sample size, our aim is not to determine which techniques should or should not be used for any given trial – noting that factors other than sample size alone need to be taken into account when choosing an imaging technique – but to provide the clinical trials community with robust sample size estimates for trials based on contemporary techniques; and in the absence of a ground truth, to provide the imaging community with a means of comparison. To this end the MIRIAD dataset – in both a blinded (challenge) and unblinded (ordered) form – will remain publicly available at http://www.ucl.ac.uk/drc/research/miriad-scan-database for the community to continue to evaluate their methods. The statistical analysis as it was performed in the challenge will also be available so that new submissions can be evaluated using the same methods.

The following are the supplementary data related to this article.

Supplementary material.

Supplementary Table Matrix showing the numbers of scans and scan pairs available for inclusion in the statistical analysis. All participants provided forward and backward measurements for each pair. The blue cells represent the number of control scans and scan pairs available, from a maximum of 23, whilst the red cells represent the AD scans and scan pairs available, from a maximum of 46. The number of scans and scan pairs that were excluded from analysis due to poor image quality is listed in brackets.

Supplementary data to this article can be found online at http://dx.doi.org/10.1016/j.neuroimage.2015.07.087.

## Figures and Tables

**Table 1:** Demographics of MIRIAD subjects.

Group	n	Age	Gender	Baseline MMSE
Control	23	69.7 ± 7.2	52%M/48%F	29.4 ± 0.8
AD	46	69.4 ± 7.1	41%M/59%F	19.2 ± 4

**Table 2:** Summary of submissions in the MIRIAD atrophy challenge.

Research centre	Submissions	Bias correction	Inter-subject registration	Standard/groupwise space	ROI method	Longitudinal registration	Image change measure
Bahçeşehir University	BAUMIP	SPM5 (Ashburner and Friston, 2005)	SPM5	SPM5	SPM5, ALVIN (Kempton et al., 2011), FIRST (Patenaude et al., 2011)	N/A	N/A
Brain image analysis	IOWA	N4 (Tustison et al., 2010)	BRAINS (Magnotta et al., 2002; Pierson et al., 2011)	In-house template	Tissue seg, ANN	N/A	N/A
CSIRO	CSIRO	Tissue seg (Van Leemput et al., 1999)	NiftyReg	Within-subject	Tissue seg, multi-atlas (Hsu et al., 2002)	N/A	N/A
Harvard MGH	FS_ORIG, FS_BETA	N3 (Sled et al., 1998)	Robust inverse consistent (Reuter et al., 2010)	Within-subject (Reuter et al., 2012)	Atlas (Dale et al., 1999; Fischl, 2012; Fischl et al., 2002)	N/A	N/A
INRIA^a^	INRIA	N3	Demons-LCC (Lorenzi et al., 2013; Vercauteren et al., 2008)	ADNI 200 HC	Loose regions	Demons-LCC	Regional flux analysis (Lorenzi et al., 2015)
Mayo Clinic	MAYO, MAYO_BSI^c^, MAYO_TBM	N3/SPM5	NiftyReg	ADNI 200 HC + 200 AD	SPM5, Seg Prop	NiftyReg 9DOF (BSI), SyN (TBM)	BSI (Gunter et al., 2003), Jacobian integration (TBM)
Montreal Neurologic Institute	MNI	ANIMAL (Collins and Evans, 1997)	ICBM152	Within-subject template	Patch-based (Coupé et al., 2011; Eskildsen et al., 2012; Fonov et al., 2012)	N/A	N/A
University College London	UCL, UCL_BSI	N3	NiftyReg (Ourselin et al., 2001)	Challenge data and template library	Multi-atlas Seg Prop (Cardoso et al., 2013)	NiftyReg (Modat et al., 2010)	DBC, Symmetric BSI (Leung et al., 2011b)
University of Pennsylvania^b^	UPENN, UPENN_DBM		FLIRT (Jenkinson and Smith, 2001) + SyN	Within-subject	Multi-atlas seg prop^d^ (Wang et al., 2012)	SyN (Avants et al., 2008)	Mesh-based (half-way space) (Yushkevich et al., 2010)

^a^ INRIA provided submissions only for the lateral ventricles and hippocampi, ^b^ University of Pennsylvania only provided submissions for the hippocampi, ^c^ the MAYO_BSI submission only included whole brain and lateral ventricle atrophy, and ^d^ template for multi atlas segmentation propagation in UPENN technique consisted of 30 randomly selected ADNI.

**Table 3:** Median (95% CI) symmetry differences by structure, group, and technique. These differences are between the forward and backward atrophy, divided by the average measures of atrophy. The UCL and INRIA measures are designed to be symmetric: thus there are no differences and they were excluded.

Group	Brain		Ventricle		Left hippocampus		Right hippocampus	
HC	AD	HC	AD	HC	AD	HC	AD
Mayo_BSI	0.0% (0.0, 0.0)	0.0% (− 0.2, 0.0)	0.0% (0.0, 0.0)	0.0% (− 0.1, 0.0)	N/A	N/A	N/A	N/A
Mayo_TBM	− 0.9% (− 3.7, 1.4)	− 1.4% (− 2.3, − 0.5)	0.0% (− 1.1, 2.7)	0.0% (− 0.4, 0.4)	− 6.0% (− 11.1, 1.7)	− 0.3% (− 2.4, 1.6)	2.3% (− 4.7, 5.2)	− 1.2% (− 3.5, 1.8)
UPenn_DBM	N/A	N/A	N/A	N/A	9.6% (− 10.4, 54.4)	3.3% (− 1.1, 8.0)	− 13.1% (− 56.5, 49.2)	− 8.5% (− 19.4, 9.9)

**Table 4:** Median (95% CI) transitivity differences by structure, group, and technique. Transitivity differences are defined to be the difference between the two 12 month atrophy measures (one coming from summing baseline and 6 months to 6 months and 12 months, and the other coming from the direct baseline to 12 months), divide by the average of these two atrophy measures.

Group	Brain		Ventricle		Left hippocampus		Right hippocampus	
HC	AD	HC	AD	HC	AD	HC	AD
INRIA	N/A	N/A	− 1.7% (− 3.4, − 0.3)	− 0.8% (− 1.4, 0.1)	1.7% (− 3.1, 8.8)	0.0% (− 1.0, 2.3)	− 0.1% (− 4.2, 5.7)	− 1.8% (− 2.9, − 0.6)
Mayo_ BSI	0.0% (− 0.3, 1.5)	0.0% (0.0, 0.0)	− 0.2% (− 0.6, 0.6)	0.2% (0.1, 0.4)	N/A	N/A	N/A	N/A
Mayo_ TBM	1.4% (− 2.3, 5.2)	− 2.2% (− 3.6, − 0.8)	0.9% (− 1.7, 2.0)	− 3.5% (− 3.8, − 2.2)	− 3.5% (− 33.8, 23.6)	− 1.5% (− 5.4, 10.2)	19.5% (− 3.8, 50.1)	− 3.2% (− 11.5, 12.5)
UCL_ BSI	0.5% (− 0.1, 1.4)	0.2% (− 0.2, 0.4)	0.1% (− 0.2, 0.2)	0.0% (0.0, 0.2)	14.9% (− 8.5, 35.4)	0.2% (− 0.8, 5.0)	0.8% (− 8.3, 17.0)	− 1.3% (− 3.7, 6.1)
UPenn_DBM	N/A	N/A	N/A	N/A	− 4.1% (− 64.0, 50.6)	− 2.8% (− 11.2, 2.9)	− 1.6% (− 42.0, 64.2)	− 9.8% (− 25.2, 4.9)

**Table 5:** Between (*σ*_*b*_^2^) and within (*σ*_*w*_^2^) components of variance over one year (95% CI) in the AD subjects by structure and technique. These can be used to compute the total variance of rates of change over any follow-up time of *t* years (*σ*_*b*_^2^ + *σ*_*w*_^2^/*t*^2^).

	Brain		Ventricle		Left hippocampus		Right hippocampus	
Between subject variance (%/year)^2^	Within subject variance (%/year)^2^	Between subject variance (%/year)^2^	Within subject variance (%/year)^2^	Between subject variance (%/year)^2^	Within subject variance (%/year)^2^	Between subject variance (%/year)^2^	Within subject variance (%/year)^2^
BAUMIP[I]	1.88 (1.34, 2.67)	1.14 (0.78, 1.70)	19.21 (11.62, 34.01)	3.58 (2.72, 4.70)	0.17 (0.00, 5.83)	38.57 (10.23,152.04)	21.57 (3.56, 55.63)	48.78 (22.38, 102.40)
CSIRO[I]	0.21 (0.12, 0.35)	0.54 (0.41, 0.71)	14.88 (10.46, 22.45)	2.85 (2.17, 4.10)	3.27 (1.54, 6.61)	9.59 (6.80, 15.74)	3.03 (1.39, 6.36)	7.77 (5.99, 10.96)
FS51ORIG[I]	0.81 (0.46, 1.62)	2.53 (1.60, 5.29)	14.85 (10.30, 23.58)	3.04 (2.29, 4.12)	3.65 (1.88, 6.84)	5.56 (4.49, 7.38)	4.47 (2.48, 8.01)	7.12 (5.69, 9.33)
FS52BETA[I]	–	–	14.36 (9.93, 23.26)	3.00 (2.25, 4.08)	3.42 (2.12, 5.41)	6.15 (4.86, 7.94)	4.90 (2.62, 8.60)	6.55 (5.29, 8.96)
INRIA[D]	–	–	3.18 (2.28, 4.92)	0.82 (0.59, 1.38)	5.84 (4.39, 8.10)	1.92 (1.47, 2.79)	5.07 (3.59, 7.23)	2.30 (1.74, 3.50)
IOWA[I]	0.77 (0.46, 1.24)	1.17 (0.76, 2.43)	10.41 (6.79, 17.46)	3.84 (3.01, 5.05)	12.26 (7.00, 21.6)	19.62 (11.85, 41.32)	19.24 (13.53, 27.73)	15.90 (10.36, 24.40)
MAYO[I]	1.29 (0.90, 1.90)	0.86 (0.69, 1.11)	14.54 (10.12, 22.28)	3.57 (2.72, 5.19)	4.90 (3.09, 8.26)	4.37 (3.58, 5.40)	10.4 (6.92, 16.18)	7.78 (6.13, 10.41)
MAYO_BSI[D]	0.96 (0.53, 1.63)	1.46 (1.02, 2.09)	13.86 (9.55, 21.08)	4.31 (3.35, 6.25)	–	–	–	–
MAYO_TBM[D]	1.01 (0.67, 1.58)	0.58 (0.46, 0.71)	12.36 (8.51, 19.51)	2.96 (2.25, 4.60)	2.19 (1.34, 3.62)	1.92 (1.55, 2.33)	2.32 (1.30, 4.22)	1.92 (1.56, 2.36)
MNI[I]	0.41 (0.21, 0.66)	0.65 (0.40, 1.15)	14.43 (9.96, 23.54)	3.12 (2.37, 4.17)	1.94 (0.85, 4.28)	5.40 (4.42, 6.58)	2.67 (1.27, 5.50)	5.54 (4.67, 6.62)
UCL[I]	0.40 (0.18, 0.76)	1.08 (0.71, 1.68)	17.17 (12.03, 26.46)	3.94 (3.02, 5.24)	4.24 (1.72, 9.73)	12.00 (9.49, 15.62)	4.60 (2.62, 7.73)	10.05 (8.24, 13.78)
UCL_BSI[D]	0.47 (0.32, 0.72)	0.19 (0.14, 0.38)	15.52 (10.91, 23.61)	2.80 (2.11, 4.31)	10.00 (6.35, 16.59)	6.93 (5.47, 9.16)	8.50 (5.08, 13.87)	7.51 (6.15, 9.46)
UPENN[I]	–	–	–	–	1.65 (0.88, 2.89)	2.53 (1.92, 3.25)	1.97 (1.10, 3.19)	1.98 (1.60, 2.71)
UPENN_DBM[D]	–	–	–	–	1.64 (0.93, 2.89)	2.61 (2.02, 3.34)	1.83 (1.04, 2.96)	2.10 (1.72, 2.75)

**Table 6:**
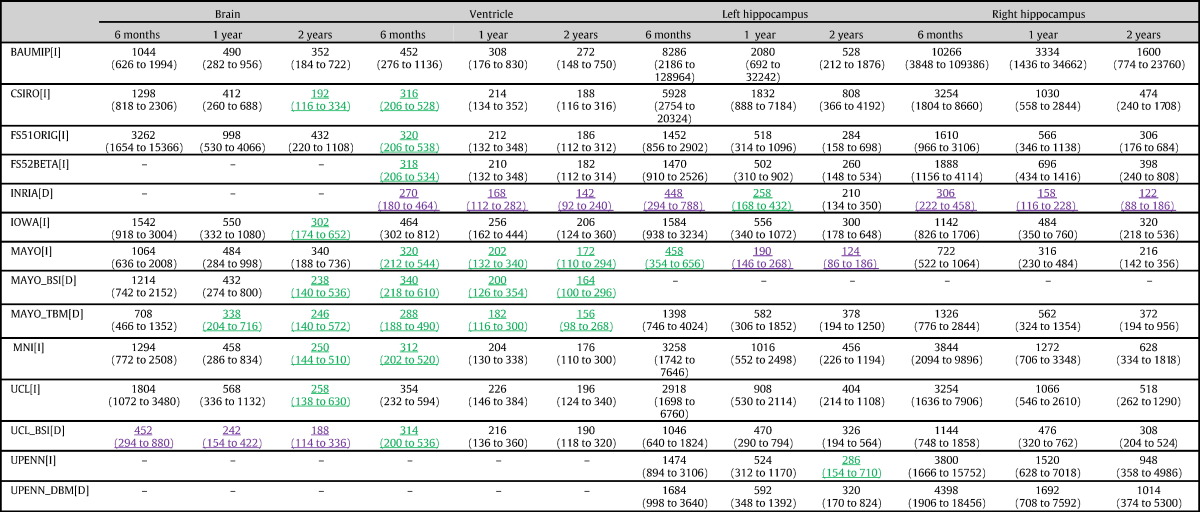
Required total sample sizes (both groups combined) for clinical trials assuming that a putative treatment can reduce the excess atrophy rate (over and above that seen in healthy controls) by 25% without altering variability. Calculations assume that the trial will have 80% statistical power to detect a treatment effect using a conventional two-sided significance level of 5%. Results shown in bold and underlined purple are the best for each structure and time interval. Those underlined and shown in green are not statistically significantly worse than best.

## Footnotes

3 http://www.ucl.ac.uk/drc/research/miriad-scan-database.
