# Supplementary material for: Assessing atrophy measurement techniques in dementia: Results from the MIRIAD atrophy challenge
Source: Neuroimage. 2015 Dec;123:149–64. doi: 10.1016/j.neuroimage.2015.07.087 (PMC4634338; doi:10.1016/j.neuroimage.2015.07.087)
Supplement: Supplementary file 1 — Supplementary material. [file mmc1.docx]

**Supplementary material: Challenge Participants Methodology**

While each group employed their own unique pipeline, there were many common steps and types of approaches that were implemented, and it will be helpful to homogenize the language between them. There were the common pre-processing steps of *bias correction*, to reduce spatially-varying intensity nonuniformities in the image, and *standard space alignment*, to re-orient the images to a consistent anatomical orientation. This orientation is often based on a well-established neuroimaging atlas or a groupwise average of the study data. After pre-processing, most of the groups segmented the key structures in each image, primarily through two main methods. The first method was a *tissue segmentation* that divides the brain into three main tissue types: grey matter (GM), white matter (WM), and cerebrospinal fluid (CSF). In some cases, the tissue segmentation is simultaneously performed with the bias correction and standard space alignment steps. Many groups summed the GM and WM components of this tissue segmentation to obtain whole brain regions of interest (ROI). The other segmentation approach commonly used was *segmentation propagation*. Here, the *unseen* image that is to be segmented is registered with one or many *template* datasets that already have the desired structure delineated, often by an expert rater. The label describing the structure is propagated from the template(s) to the unseen image. In the case of multiple templates, a *label fusion* strategy is required to determine a consensus labeling of the ROIs for the unseen image.

Change is often measured simply by computing the volume of an ROI for each time point and then taking the difference between the two results. Some participants performed a *direct measure* of change, where the change of volume was computed based on the difference between the two images, whether intensity based (such as in the boundary shift integral) or deformation based (by analyzing the deformation field required to non-linearly align between the two images).

### Bahçeşehir University (BAUMIP)

Bias correction, standard space alignment, and tissue segmentation are performed using the unified segmentation framework (Ashburner and Friston, 2005) that is part of the SPM (Statistical Parametric Mapping) package^[[1]](#footnote-1)^ . Ventricle segmentation was performed with Automatic Lateral Ventricle delIneatioN (ALVIN) (Kempton et al., 2011), an SPM8 extension. Finally, hippocampal segmentation was performed using the FSL package FIRST (Patenaude et al., 2011), which uses a Bayesian shape and appearance model to segment subcortical structures. The participants added a constraint on the measures, such that any measurement from a blinded time point could not have a volume greater (or in the cases of ventricles, less) than the identified baseline, ensuring a change of zero for these instances.

### Brain Image Analysis (Iowa)

This submission uses an automated image-processing pipeline (Pierson et al., 2011) based on the BRAINS (Magnotta et al., 2002) software package. Typically, this package expects multimodal data from T1 and T2 images, but for the MIRIAD atrophy challenge, it was adapted to handle T1 data only. The steps are still quite similar to (Pierson et al., 2011): a multi-step standard space alignment (Johnson et al., 2007) to a groupwise template, followed by bias correction using the N4 algorithm (Tustison et al., 2010). Tissue segmentations were refined using a discriminant classifier, and hippocampal segmentation was performed using an artificial neural network (ANN) algorithm.

### CSIRO

An in-house tissue segmentation method based on (Van Leemput et al., 1999) was first performed. The resulting tissue masks were used to skull-strip the T1W images. Unbiased within-subject templates were then created with a procedure similar to that of FreeSurfer (Reuter 2012). The tissue probability maps were propagated to the mean within-subject template space, and averaged across all timepoints to generate subject specific priors. Each image was then segmented in the mean space using the subject specific priors to generate the final tissue segmentations. The within-subject templates were parcellated by segmentation propagation using the NiftyReg package^[[2]](#footnote-2)^ (Modat et al., 2010) for the non-rigid registration. Each unseen timepoint was also parcellated in this manner, initializing the non rigid registration with the deformation fields computed from the within-subject template. Using this first whole brain parcellation, a region of interest was defined around the hippocampus, and the procedure was reiterated within the ROI to obtain the final hippocampus parcellation. The final parcellations were masked using the GM segmentation. The anatomical definition of the hippocampus for the template was based on the semi-automatic segmentation generated using the Surgical Navigation Technologies (SNT) software (Hsu et al., 2002)on the ADNI database.

### Harvard/Mass General Hospital (FreeSurfer)

All images were processed using the longitudinal pipeline of FreeSurfer (Dale et al., 1999; Fischl, 2012; Fischl et al., 1999a, 1999b; Reuter et al., 2012). First all time points are skull stripped and bias corrected. They are then used to create a within-subject template by performing an iterative robust, inverse consistent rigid registration (Reuter et al., 2010) to an average space, which is based on voxelwise intensity median rather than the mean to reduce the effect of outliers. The image segmentation as well as pial and white matter surface models are constructed on the within-subject template. The surfaces serve as an initialization for the surface construction on each individual time point. Initial subcortical segmentations are performed by non-linearly registering each image to a probabilistic atlas, which consists of 40 subjects of varying age and Alzheimer's pathology. In some cases, an additional special flag is used to increase flexibility and segmentation accuracy for subjects with very large ventricles. After initial segmentations are available, the final image segmentation of each time point is performed in the subject template space via label fusion incorporating information across all time points. An additional run (FS 5.2 beta) was submitted, where a cubic interpolation was used instead of trilinear, and the extra processing step to account for subjects with large ventricles was not performed. In the meantime FS 5.2 has been shown to produce inaccurate pial and white matter surfaces, potentially affecting the full brain measurements (this has been resolved in version 5.3). For the challenge, the left and right hippocampal volumes are summed. The ventricle volume is represented by left and right lateral and inferior lateral ventricle, and includes choroid plexus. The full brain measure consists of the partial volume of all labelled structures after surface correction, including vessel and optic-chiasm, but without brainstem, ventricles (lateral, inferior lateral, 3rd, 4th, 5th), CSF and choroid plexus.

### INRIA Asclepios/Fatebenefratelli (INRIA)

This submission uses a regional flux analysis (Lorenzi et al., 2012) to determine percentage change between each pair of scans for ventricles and hippocampi. Before performing the flux analysis, all images in the time series are aligned to the baseline using a 9 DOF linear transformation, refining the registration based on results from skull stripping (Iglesias and Thompson, 2011). A standard space alignment is then performed on the aligned time-series using a 12 DOF affine transformation to an atlas created from a subset of healthy elderly subjects from the ADNI cohort. All linear registrations were performed by FLIRT (Jenkinson and Smith, 2001) and combined into one interpolation step. Non-rigid registrations between baseline and follow-up images were performed using the LCC-Demons method(Lorenzi et al., 2013), which is an extension of the symmetric log demons algorithm (Vercauteren et al., 2008) using a local cross-correlation as the similarity metric. From the stationary velocity fields obtained in the non-rigid registration, the longitudinal atrophy is modeled by obtaining the flux of the velocity field at the region boundary. Inward flow across a surface represents compression, or atrophy, while outward flow represents expansion. Volume change is calculated by integrating the divergence of the velocity field over the volume. Unlike the other methods, which attempt to delineate a precise boundary around the structure of interest, this method uses ventricular and hippocampal regions loosely defined around both the anatomic information and priori information of atrophy in AD, estimated from a subset of AD patients in the ADNI cohort (Lorenzi et al., 2011). These probabilistic regions were defined in the template space and propagated to each image using non-rigid registration.

### Mayo Clinic

Three separate methods were submitted: one using volumetric differences and two using direct measures of change. The first preprocessing step in all three submissions was standard space alignment to a template of 400 subjects (200 controls, 200 AD) aligned to the MNI coordinate system. Next, the image was segmented using SPM5 to create a total intracranial volume (TIV) mask, which was used for bias correction using N3 (Sled 1998). SPM5 was then re-run on the bias corrected images. The resulting brain mask was cleaned up through a region-growing algorithm that started in voxels with high GM and WM probabilities. Lateral ventricles and hippocampi were delineated using segmentation propagation from the template image, refining the results with the corresponding tissue properties at each voxel. Pairwise rigid registration was performed between the identified baseline scan and all the follow-up scans for each subject using a rigid registration based on the block matching algorithm (Ourselin et al., 2001) publicly available in the NiftyReg package. The registration was limited to the region of the TIV mask. The pairwise transformations were combined to create a within-subject template space, and all images and masks were transformed and resampled into this template space at 1mm isotropic resolution. The final step was to create an average image and mask from these transformed images. As the BSI method is essentially an intensity subtraction, the image intensities need be well-matched. Using morphological operators on the automatically delineated brain mask regions dominated by CSF, grey matter, and white matter were determined.   For each image Gaussian distributions were fitted to intensities in white matter and CSF spatial regions.  Image intensities are linearly scaled so that the CSF and white matter peaks were at 5,000 and 20,000 arbitrary units.  A single subject mean image was calculated as the average of the intensity balanced images after spatial co-registration.  The Gaussian fits were repeated for the mean image. In addition to white matter and CSF intensities estimates, a grey matter intensity estimate is formed by fitting a sum of two Gaussian distributions to regions expected to primarily contain grey matter. One of the distributions has a center and width fixed at the values obtained from the respective white matter region fit.   Images intensities in the individual time point images are adjusted by a spline-based remapping to bring the CSF, grey matter and white matter intensities estimates in lines with those of the mean image.  A differential bias correction is then performed to reduce residual shading artifacts for each input image relative to the mean image.  Voxels which are within two standard deviations of the gaussian fitted centroids for CSF or white matter in both the mean image and each individual image are selected.  The log of the ratio of those (scattered) voxels in the individual image to the mean image is formed.  Additionally, the outer edges of the log-ratio image are set to zero.  A dense field is obtained by tri-linear 3D-interpolation of the defined points in the log-ratio image.  The dense field is then smoothed with a gaussian kernel with width 20mm.  The resulting field is exponentiated and multiplicatively applied to the individual image. The boundary shift integral (BSI; Gunter et al., 2003) was computed for the whole brain and ventricle regions, with parameters optimized for this data set to find values most consistent with volumetric differences. The second method of direct change was based on non-rigid registration using the symmetric normalization (SyN) algorithm (Avants et al., 2008), producing deformation fields in both directions for each time-point combination. The Jacobians from the resulting deformation field were computed within the brain, ventricle, and hippocampal masks to obtain volume change.

### Montreal Neurological Institute (MNI)

The first pre-processing step is a denoising filter using a patch based method (Coupe et al., 2008), followed by bias correction using N3 (Sled et al., 1998), and standard space alignment to ICBM152 template space (Collins et al., 1994). A within-subject template is created using an iterative groupwise approach: first an affine transformation with cross-correlation as the similarity metric, then a non-linear registration step using ANIMAL (Collins and Evans, 1997). At the end of each iteration, the template is created by averaging the images as well as correcting for inter-visit inhomogeneity using a method similar to DBC. For the non-linear registrations, the parameters are modified with increasing iterations in a coarse to fine strategy. Segmentation of all structures are performed on the template with a patch based method (Coupé et al., 2011; Eskildsen et al., 2011; Fonov et al., 2012) using a template that contains manual segmentations labeled by experts. The resulting segmentations are then propagated from the subject template to each time-point using the deformation fields constructed during the template construction.

### University College London (UCL)

Bias correction is performed on all images using the N3 algorithm (Sled et al., 1998), followed by a groupwise affine registration, using a block matching algorithm (Ourselin et al., 2001). In this case, the groupwise is created not only with all of the data in the MIRIAD atrophy challenge, but also with the template library used for segmentation. The template library consisted of 89 T1-weighted scans that had manual segmentations of the brain and ventricles and 66 scans that had left and right hippocampal segmentations. It should be noted that the template library did not include any scans and segmentations from the MIRIAD study. All images and their associated labels were flipped in the left-right direction to double the number of images in the template library. Segmentation was performed using a multi-atlas segmentation propagation technique. All template images were non-linearly registered to the unseen image using a free form deformation model in the NiftyReg package (Modat 2010) with normalized mutual information as a similarity metric. The propagated labels were then fused using STEPS (Cardoso 2013), a modification to the STAPLE technique, where at each voxel, only the most similar template images, as determined by the locally normalized cross-correlation, are included for the label fusion. A Markov random field is added to the label fusion process in order to provide better spatial consistency of the labeling. After segmentation, longitudinal change is assessed using a symmetric groupwise implementation of differential bias correction and the boundary shift integral (Leung et al., 2011).

### University of Pennsylvania (UPenn)

Our system contains two sequential components: 1) producing segmentations for a structure of interest in testing images; 2) estimating volume change for the structure of interest. For volume change estimation, both linear registration and deformable registration are estimated using the latest version of ANTs software (picsl.upenn.edu/ants), which is implemented based on the Version 4 of the Insight Toolkit (ITK). We describe each component in detail below. Note that the processing decisions made for this challenge were impacted by time constraints and may not be optimal in all cases.

*Segmentation of the structure of interest*

Our study focuses on measuring atrophy for the hippocampus. To allow pairwise atrophy measurements between any two time points for the hippocampus, for each subject, we produce segmentation for the hippocampus at each time point using multi-atlas label fusion with 30 randomly selected atlases from the ADNI 1.5 Tesla dataset.

To apply the atlas-based approach for segmenting the hippocampus, image-based registration was performed in two steps: a global affine registration and a deformable registration. We follow the registration pipeline implemented by AHEAD (http://www.nitrc.org/projects/ahead/; Hanson et al., 2012), which is optimized for segmenting the hippocampus. Global affine registration is performed using the FSL FLIRT tool (Jenkinson and Smith, 2001) with the default parameters. Based on the global affine registration, deformable registration is performed using the greedy diffeomorphic Symmetric Normalization (SyN) algorithm implemented by ANTS (Avants et al., 2008). SyN registrations used the cross-correlation metric with a 5 x 5 x 5 window; 3 resolution levels with maximum 80, 20 and 5 iterations at the coarse, middle and fine levels, respectively; step size 0.25; Gaussian regularization with standard deviation of 3 pixels. After registration, reference segmentations from each of the atlases were warped into the target image space.

For each hippocampus, 30 candidate segmentations were produced by warping the labels from the atlases. The final segmentation is obtained by combining the candidate segmentations with joint label fusion (Wang et al., 2012). The joint

label fusion algorithm has three parameters: *r*, the radius of the local appearance window $\mathcal{N}$ used in similarity-based pairwise dependency estimation; *r_s_*, the radius of the local searching window $\mathcal{N'}$ used in remedying registration errors; and $\beta$, the parameter used to transfer image similarities to dependency estimation. For this study, we apply (*r*, *r_s_*, $\beta$) = (2,3,2) to segment all hippocampi, which was shown in our previous study (Wang et al., 2012) to be optimal for segmenting the hippocampus.

*Processing pipeline for longitudinal change measurements*

Each image underwent intensity inhomogeneity correction using the N4ITK tool (Tustison et al., 2010). To measure the longitudinal change in the hippocampus between two time points, we applied 9-parameter linear registration that accounts for possible anisotropic scaling sometimes present due to scanner drift, in addition to rigid rotation and translation. Instead of registering the whole brain images, we registered a pair of small ROI images around the hippocampi. These ROIs were defined by dilating the hippocampus segmentation mask by 5 voxels and including 10 additional voxels beyond the boundary of the dilated mask. This was done separately for left and right hippocampi. The "Similarity" transformation model as implemented in the latest version of ANTs software, based on ITKv4, was used. The Mattes mutual information metric is applied with 32 bins, the regular sampling strategy and 0.05 sampling percentage. Three resolution levels with maximum 200, 200 and 50 iterations at the coarse, middle and fine levels, respectively. These parameters were selected via optimization on the ADNI longitudinal dataset and for speed.

As has been discussed extensively in the literature (Fox et al., 2011; Reuter et al., 2010; Yushkevich et al., 2010), to ensure unbiased longitudinal measurements, it is important to compute the image similarity metric $\Pi$ in a symmetric fashion, so that both images undergo the same, minimal number of resampling operations, as well as the same amount of global transformation before applying the deformable transformations $\phi_{1}$ and $\phi_{2}$ and measuring similarity between them. Let $R_{ref}\left( I,\psi\right)$ define a resampling operator that produces an image *I’*, which is resampled in the space of a reference image *I_ref_* , from an image *I* after applying a spatial transformation $\psi$ .

If *I* and *I_ref_* are defined on a lattice of points $\left\{ x_{i} \right\}\in\Omega$ and $\left\{ y_{j} \right\}\in\Omega_{ref}$ respectively, we have

$$I^{'\left( y_{j} \right)}=\sum_{i} \mathcal{L}\left( \psi\left( y_{j} \right)-x_{i} \right)I\left( x_{i} \right)$$

where $\mathcal{L}$ is an interpolation kernel. We use a tent function which corresponds to linear interpolation. If *M* is the derived global transformation matrix between the baseline and followup images *I^BL^* and *I^FU^* respectively, following (Yushkevich et al., 2010), symmetric computation of $\Pi$ is given by

$$\Pi\left[ R_{ref}\left( I^{BL},M^{-\frac{1}{2}} \circ\phi_{1} \right),R_{ref}\left( I^{FU},M^{\frac{1}{2}} \circ\phi_{2} \right) \right]$$

where $\circ$ represents the composition operator.

Deformable registration is performed using the SyN algorithm (Avants et al., 2010)[1] implemented by the latest ANTs software. For maximal sensitivity in longitudinal registration scenarios, we chose the following parameters. The Mattes mutual information image similarity metric with a bin size of 32 is used for the deformable registration. Three resolution levels with maximum 1200, 1200 and 100 iterations at the coarse, middle and _ne levels, respectively. Other parameters are gradient step = 0.25, update field variance $\sigma$ = 2, total field variance $\sigma$ = 0.5. The input images themselves are not smoothed before metric computation.

Traditional DBM uses Jacobian determinant of the deformation field to estimate local change. However, its computation requires computing spatial derivatives of $\phi$, which is computed in the discrete domain using finite difference approximation. This mixes information from a number of neighboring voxels. Further, to avoid numerical errors, the deformation field needs to be very smooth. Since we are interested in subtle changes over narrow regions, we wanted to limit unnecessary smoothing of the deformation field, and cross-contamination from adjacent ROIs. This motivated us to consider an alternative, more direct, way of measuring atrophy using a simple mesh-based approach as we did in our previous work (Yushkevich et al., 2010). We fit a volumetric mesh consisting of tetrahedral elements to each ROI label in the baseline image. The sum of volumes of all the mesh elements is taken as the volume before applying the deformation *V_BD_*. We apply the 3D deformation field to each vertex of the mesh and recompute and add the volumes after deformation *V_AD_*. The volume change is measured as the volume difference before and after applying the deformation field, i.e. *V_BD_* - *V_AD_*. The TetGen (http://tetgen.berlios.de) software package is used for 3D mesh generation.

Since the segmentation of each time point is performed independently, the differences between volumes at different timepoints are likely to be dominated by segmentation error, and do not take into account pairwise volume change measurements computed by DBM. In order to produce volume measurements that take into account the DBM atrophy measurements, we employ the following general linear model for each subject:

| $0=\left( 1-a_{ij} \right)\omega_{i}-\omega+\beta+ \epsilon_{ij}$ | $\forall i,j\in\left\{ 1,n \right\} s.t. i\neq j ,$ |
| --- | --- |
| $\lambda V_{k}=\lambda\omega_{k}+\eta_{k}$ | $\forall k\in\left\{ 1,n \right\},$ |

where *n* is the number of timepoints for the subject; $\omega_{1}\ldots\omega_{n}$ are the unknown volume measurements to be estimated at each timepoint; *a_ij_* is the relative atrophy computed by DBM in an experiment with *i*-th timepoint as the baseline image and *j*-th timepoint as the follow-up image; $\beta$ is an unknown parameter accounting for bias in volume estimation, and $\epsilon_{ij}$ ; $\eta_{k}$ are independent identically distributed univariate normal random variables with mean 0; and $\lambda$ is a weighting factor discussed below. Informally, the first part of the model specifies that every pair of volume measurements $\left\{ \omega_{i},\omega_{j} \right\}$ should conform to the relative atrophy measurement from the DBM experiment, and the second part of the model specifies that these volumes should not deviate too much from the volumes computed by multi-atlas segmentation. The factor $\lambda$ weights the relative importance of these components of the model. The model is solved using the ordinary least squares approach. Robust regression methods may also be employed to help reduce the influence of outlier relative atrophy measurements. However, our preliminary examination of the data did not reveal substantial effects of outliers and robust regression was not used. The factor $\lambda$ was set to 0.1, although we found that varying $\lambda$ by an order of magnitude had only a minor effect on the estimated volumes.

**References**

Ashburner, J., Friston, K.J., 2005. Unified segmentation. Neuroimage 26, 839–51.

Avants, B.B., Epstein, C.L., Grossman, M., Gee, J.C., 2008. Symmetric diffeomorphic image registration with cross-correlation: evaluating automated labeling of elderly and neurodegenerative brain. Med. Image Anal. 12, 26–41.

Avants, B.B., Yushkevich, P., Pluta, J., Minkoff, D., Korczykowski, M., Detre, J., Gee, J.C., 2010. The optimal template effect in hippocampus studies of diseased populations. Neuroimage 49, 2457–66.

Collins, D.L., Evans, A.C., 1997. Animal: Validation and Applications of Nonlinear Registration-Based Segmentation. Int. J. Pattern Recognit. Artif. Intell. 11, 1271–1294.

Collins, D.L., Neelin, P., Peters, T.M., Evans, A.C., 1994. Automatic 3D Intersubject Registration of MR Volumetric Data in Standardized Talairach Space. J. Comput. Assist. Tomogr. 18, 192–205.

Coupé, P., Eskildsen, S.F., Manjón, J. V, Fonov, V., Collins, D.L., 2011. Simultaneous segmentation and grading of anatomical structures for patient’s classification: Application to Alzheimer's disease. Neuroimage 59, 3736–3747.

Coupe, P., Yger, P., Prima, S., Hellier, P., Kervrann, C., Barillot, C., 2008. An optimized blockwise nonlocal means denoising filter for 3-D magnetic resonance images. IEEE Trans. Med. Imaging 27, 425–41.

Dale, A.M., Fischl, B., Sereno, M.I., 1999. Cortical Surface-Based Analysis I: Segmentation and Surface Reconstruction. Neuroimage 9, 179–194.

Eskildsen, S.F., Coupé, P., Fonov, V., Manjón, J. V, Leung, K.K., Guizard, N., Wassef, S.N., Ostergaard, L.R., Collins, D.L., 2011. BEaST: Brain extraction based on nonlocal segmentation technique. Neuroimage.

Fischl, B., 2012. FreeSurfer. Neuroimage 62, 774–81.

Fischl, B., Sereno, M.I., Dale, A.M., 1999a. Cortical Surface-Based Analysis II: Inflation, Flattening, and a Surface-Based Coordinate System. Neuroimage 9, 195–207.

Fischl, B., Sereno, M.I., Tootell, R.B.H., Dale, A.M., 1999b. High-resolution intersubject averaging and a coordinate system for the cortical surface. Hum. Brain Mapp. 8, 272–84.

Fonov, V.S., Coupé, P., Styner, M., Collins, D.L., 2012. Automatic lateral ventricle segmentation in infant population with high risk of autism, in: Annual Meeting of the Organisation for Human Brain Mapping. Beijing, China.

Fox, N.C., Ridgway, G.R., Schott, J.M., 2011. Algorithms, atrophy and Alzheimer’s disease: cautionary tales for clinical trials. Neuroimage 57, 15–8.

Gunter, J.L., Shiung, M.M., Manduca, A., Jack, C.R., 2003. Methodological considerations for measuring rates of brain atrophy. J. Magn. Reson. Imaging 18, 16–24.

Hanson, J.L., Suh, J.W., Nacewicz, B.M., Sutterer, M.J., Cayo, A. a., Stodola, D.E., Burghy, C. a., Wang, H., Avants, B.B., Yushkevich, P. a., Essex, M.J., Pollak, S.D., Davidson, R.J., 2012. Robust automated amygdala segmentation via multi-atlas diffeomorphic registration. Front. Neurosci. 6, 1–8.

Hsu, Y.Y., Schuff, N., Du, A.T., Mark, K., Zhu, X., Hardin, D., Weiner, M.W., 2002. Comparison of automated and manual MRI volumetry of hippocampus in normal aging and dementia. J. Magn. Reson. Imaging 16, 305–310.

Iglesias, J.E., Thompson, P.M., 2011. Robust Brain Extraction Across Datasets and Comparison With Publicly Available Methods. IEEE Trans. Med. Imaging 30, 1617–1634.

Jenkinson, M., Smith, S., 2001. A global optimisation method for robust affine registration of brain images. Med. Image Anal. 5, 143–56.

Johnson, H., Harris, G., Williams, K., Johnson, H.J., Harris, G., Pieper, S., 2007. BRAINSFit : Mutual Information Rigid Registrations of Whole-Brain 3D Images , Using the Insight Toolkit. Insight J. 1–10.

Kempton, M.J., Underwood, T.S. a, Brunton, S., Stylios, F., Schmechtig, A., Ettinger, U., Smith, M.S., Lovestone, S., Crum, W.R., Frangou, S., Williams, S.C.R., Simmons, A., 2011. A comprehensive testing protocol for MRI neuroanatomical segmentation techniques: Evaluation of a novel lateral ventricle segmentation method. Neuroimage 58, 1051–9.

Leung, K.K., Ridgway, G.R., Ourselin, S., Fox, N.C., 2011. Consistent multi-time-point brain atrophy estimation from the boundary shift integral. Neuroimage 59, 3995–4005.

Lorenzi, M., Ayache, N., Frisoni, G.B., Pennec, X., 2011. Mapping the Effects of Aβ 1 − 42 Levels on the Longitudinal Changes in Healthy Aging : Hierarchical Modeling Based on Stationary Velocity Fields, in: Medical Image Computing and Computer-Assisted Intervention (MICCAI2011), Volume 6892 of Lecture Notes in Computer Science. pp. 663–670.

Lorenzi, M., Ayache, N., Frisoni, G.B., Pennec, X., 2013. LCC-Demons: a robust and accurate symmetric diffeomorphic registration algorithm. Neuroimage 81, 470–83.

Lorenzi, M., Ayache, N., Pennec, X., 2012. Regional Flux Analysis of Longitudinal Atrophy in Alzheimer ’ s Disease, in: Medical Image Computing and Computer-Assisted Intervention (MICCAI2012), Volume 7510 of Lecture Notes in Computer Science. pp. 739–746.

Magnotta, V. a, Harris, G., Andreasen, N.C., O’Leary, D.S., Yuh, W.T.C., Heckel, D., 2002. Structural MR image processing using the BRAINS2 toolbox. Comput. Med. Imaging Graph. 26, 251–64.

Modat, M., Ridgway, G.R., Taylor, Z. a., Lehmann, M., Barnes, J., Hawkes, D.J., Fox, N.C., Ourselin, S., 2010. Fast free-form deformation using graphics processing units. Comput. Methods Programs Biomed. 98, 278–284.

Ourselin, S., Roche, A., Subsol, G., Pennec, X., Ayache, N., 2001. Reconstructing a 3D structure from serial histological sections. Image Vis. Comput. 19, 25–31.

Patenaude, B., Smith, S.M., Kennedy, D.N., Jenkinson, M., 2011. A Bayesian model of shape and appearance for subcortical brain segmentation. Neuroimage 56, 907–22.

Pierson, R., Johnson, H., Harris, G., Keefe, H., Paulsen, J.S., Andreasen, N.C., Magnotta, V. a, 2011. Fully automated analysis using BRAINS: AutoWorkup. Neuroimage 54, 328–36.

Reuter, M., Rosas, H.D., Fischl, B., 2010. Highly accurate inverse consistent registration: a robust approach. Neuroimage 53, 1181–96.

Reuter, M., Schmansky, N.J., Rosas, H.D., Fischl, B., 2012. Within-subject template estimation for unbiased longitudinal image analysis. Neuroimage 61, 1402–18.

Sled, J.G., Zijdenbos, a P., Evans, a C., 1998. A nonparametric method for automatic correction of intensity nonuniformity in MRI data. IEEE Trans. Med. Imaging 17, 87–97.

Tustison, N.J., Avants, B.B., Cook, P. a, Zheng, Y., Egan, A., Yushkevich, P. a, Gee, J.C., 2010. N4ITK: improved N3 bias correction. IEEE Trans. Med. Imaging 29, 1310–20.

Van Leemput, K., Maes, F., Vandermeulen, D., Suetens, P., 1999. Automated model-based tissue classification of MR images of the brain. IEEE Trans. Med. Imaging 18, 897–908.

Vercauteren, T., Pennec, X., Perchant, A., Ayache, N., 2008. Symmetric Log-Domain Diffeomorphic Registration : A Demons-Based Approach, in: Medical Image Computing and Computer-Assisted Intervention (MICCAI2008), Part I, Volume 5241 of Lecture Notes in Computer Science. pp. 754–761.

Wang, H., Suh, J.W., Das, S.R., Pluta, J., Craige, C., Yushkevich, P. a, 2012. Multi-Atlas Segmentation with Joint Label Fusion. IEEE Trans. Pattern Anal. Mach. Intell. 35, 611–623.

Yushkevich, P. a, Avants, B.B., Das, S.R., Pluta, J., Altinay, M., Craige, C., 2010. Bias in estimation of hippocampal atrophy using deformation-based morphometry arises from asymmetric global normalization: an illustration in ADNI 3 T MRI data. Neuroimage 50, 434–45.

1. http://www.fil.ion.ucl.ac.uk/spm/ [↑](#footnote-ref-1)
2. <http://sourceforge.net/projects/niftyreg/> [↑](#footnote-ref-2)
