# Supplementary material for: Assessing atrophy measurement techniques in dementia: Results from the MIRIAD atrophy challenge
Source: Neuroimage. 2015 Dec;123:149–64. doi: 10.1016/j.neuroimage.2015.07.087 (PMC4634338; doi:10.1016/j.neuroimage.2015.07.087)
Supplement: Supplementary Table — Matrix showing the numbers of scans and scan pairs available for inclusion in the statistical analysis. All participants provided forward and backward measurements for each pair. The blue cells represent the number of control scans and scan pairs available, from a maximum of 23, whilst the red cells represent the AD scans and scan pairs available, from a maximum of 46. The number of scans and scan pairs that were excluded from analysis due to poor image quality is listed in brackets. [file mmc2.docx]

Supplemental Table - Matrix showing the numbers of scans and scan pairs available for inclusion in the statistical analysis. All participants provided forward and backward measurements for each pair. The blue cells represent the number of control scans and scan pairs available, from a maximum of 23, while the red cells represent the AD scans and scan pairs available, from a maximum of 46. The number of scans and scan pairs that were excluded from analysis due to poor image quality are listed in brackets.

|  | | Follow-up (weeks) | | | Baseline | | 2 | 6 | | 12 | 26 | 38 | | 52 | 78 | 104 |
| --- | --- | --- | --- | --- | --- | --- | --- | --- | --- | --- | --- | --- | --- | --- | --- | --- |
|  |  | Scan (1 or 2) | | | 1 | 2 | 1 | 1 | 2 | 1 | 1 | 1 | 2 | 1 | 1 | 1 |
| Follow-up (weeks) | Scan  (1 or 2) |  | | | Scans | | | | | | | | | | | |
|  |  |  |  |  | 23 | 22 | 22 | 22 | 22 | 22 | 22 | 22 | 22 | 23 | 13(1) | 8 |
|  |  |  |  |  | Scan Pairs | | | | | | | | | | | |
| Baseline | 1 | Scans | 45(3) | Scan Pairs |  | 22 | 22 | 22 | 22 | 22 | 22 | 22 | 22 | 23 | 13(1) | 8 |
|  | 2 |  | 43(1) |  | 43(3) |  | 21 | 21 | 21 | 21 | 21 | 21 | 21 | 22 | 12(1) | 7 |
| 2 | 1 |  | 44 |  | 44(3) | 42(1) |  | 21 | 21 | 21 | 21 | 21 | 21 | 22 | 13(1) | 8 |
| 6 | 1 |  | 45(1) |  | 44(3) | 42(1) | 43 |  | 22 | 21 | 21 | 21 | 21 | 22 | 12(1) | 7 |
|  | 2 |  | 41 |  | 41(2) | 40(1) | 40 | 41 |  | 21 | 21 | 21 | 21 | 22 | 12(1) | 7 |
| 12 | 1 |  | 46(2) |  | 45(5) | 43(2) | 44(2) | 45(3) | 41(1) |  | 22 | 22 | 22 | 22 | 13(1) | 8 |
| 26 | 1 |  | 44 |  | 43(3) | 41(1) | 43 | 43(1) | 40 | 44(2) |  | 22 | 22 | 22 | 13(1) | 8 |
| 38 | 1 |  | 38 |  | 38(3) | 36(1) | 38 | 37 | 34 | 38(1) | 37 |  | 22 | 22 | 13(1) | 8 |
|  | 2 |  | 35 |  | 35(2) | 34(1) | 35 | 34 | 33 | 35 | 34 | 35 |  | 22 | 13(1) | 8 |
| 52 | 1 |  | 44(1) |  | 43(3) | 41(1) | 43 | 43(1) | 39 | 44(3) | 43(1) | 38 | 35 |  | 13(1) | 8 |
| 78 | 1 |  | 26 |  | 26(2) | 25(1) | 26 | 25 | 25 | 26 | 26 | 26 | 26 | 26 |  | 8 |
| 104 | 1 |  | 14 |  | 14 | 13 | 14 | 13 | 13 | 14 | 14 | 13 | 13 | 14 | 13 |  |
